# Effects of protein-protein interactions and ligand binding on the ion permeation in KCNQ1 potassium channel

**DOI:** 10.1371/journal.pone.0191905

**Published:** 2018-02-14

**Authors:** Horia Jalily Hasani, Aravindhan Ganesan, Marawan Ahmed, Khaled H. Barakat

**Affiliations:** 1 Faculty of Pharmacy and Pharmaceutical Sciences, University of Alberta, Edmonton, Alberta, Canada; 2 Li Ka Shing Institute of Virology, University of Alberta, Edmonton, Alberta, Canada; 3 Li Ka Shing Applied Virology Institute, University of Alberta, Edmonton, Alberta, Canada; University of Cambridge, UNITED KINGDOM

## Abstract

The voltage-gated KCNQ1 potassium ion channel interacts with the type I transmembrane protein minK (KCNE1) to generate the slow delayed rectifier (I_Ks_) current in the heart. Mutations in these transmembrane proteins have been linked with several heart-related issues, including long QT syndromes (LQTS), congenital atrial fibrillation, and short QT syndrome. Off-target interactions of several drugs with that of KCNQ1/KCNE1 ion channel complex have been known to cause fatal cardiac irregularities. Thus, KCNQ1/KCNE1 remains an important avenue for drug-design and discovery research. In this work, we present the structural and mechanistic details of potassium ion permeation through an open KCNQ1 structural model using the combined molecular dynamics and steered molecular dynamics simulations. We discuss the processes and key residues involved in the permeation of a potassium ion through the KCNQ1 ion channel, and how the ion permeation is affected by (i) the KCNQ1-KCNE1 interactions and (ii) the binding of chromanol 293B ligand and its derivatives into the complex. The results reveal that interactions between KCNQ1 with KCNE1 causes a pore constriction in the former, which in-turn forms small energetic barriers in the ion-permeation pathway. These findings correlate with the previous experimental reports that interactions of KCNE1 dramatically slows the activation of KCNQ1. Upon ligand-binding onto the complex, the energy-barriers along ion permeation path are more pronounced, as expected, therefore, requiring higher force in our steered-MD simulations. Nevertheless, pulling the ion when a weak blocker is bound to the channel does not necessitate high force in SMD. This indicates that our SMD simulations have been able to discern between strong and week blockers and reveal their influence on potassium ion permeation. The findings presented here will have some implications in understanding the potential off-target interactions of the drugs with the KCNQ1/KCNE1 channel that lead to cardiotoxic effects.

## Introduction

The cardiac KCNQ1 is a voltage-gated potassium ion channel that is expressed in different tissues throughout the human body, including heart, brain, epithelia and smooth muscles [1]. In particular, KCNQ1 is involved in shaping the cardiac action potential in the heart, thus KCNQ1 is important for the normal functioning of the heart. In the cardiac myocytes, the KCNQ1 channel complexes with its beta-subunit, the transmembrane KCNE1 (minK) protein to constitute the slow component of the delayed rectifier current (I_KS_) [2]. This is mainly facilitated by allowing selective permeation of the potassium ions from the intracellular membrane to the extracellular environment, through the KCNQ1 channel [3,4]. The maintenance of this normal ion flux gives the KCNQ1/KCNE1 ion channel its unique role in controlling the duration of the repolarization phase of the cardiac action potential. Mutations in either of these proteins (i.e., KCNQ1 or KCNE1) are shown to be linked with congenital long QT syndrome (LQTS1), atrial fibrillation, and short QT syndrome [5–7]. In particular, a list of all known LQTS1-associated single-point mutations in human KCNQ1 channel are provided in S1 Table.

It has been reported in the literature that the association of these two proteins slows the activation of KCNQ1 by 5- to 10-folds [8–10]. Another profound effect of the KCNE1 protein on KCNQ1 is the paradoxical slowing of the gating associated with KCNQ1 channel inactivation, which otherwise takes place in a fast manner [11]. However, the molecular bases behind the KCNQ1/KCNE1 interactions and their impacts on the function of KCNQ1 have not been understood well. The experimental structures of either human KCNQ1 and/or human KCNQ1/KCNE1 complex have not been reported till today. The most recent experimental structure for the KCNQ1 channel is for frog species and was reported by Sun & Mackinnon in 2017 [12]. Hence, computational modelling approaches have been routinely employed to bridge the gap with experiments and to gain some insights about the structures and dynamics of KCNQ1 channel. For example, Kang et al [13] determined the experimental structure of KCNE1 using solution NMR and subsequently they used experimentally-restrained molecular docking of the transmembrane domain of a KCNQ1 potassium channel with KCNE1 and reported that the latter modulates the KCNQ1 function [13]. In another study, Xu et al [14] employed a combination of comparative modelling, protein-protein docking and molecular dynamics methods to construct the three dimensional structure of the KCNQ1/KCNE1 complex. This study [14] showed that the interactions of KCNE1 with that of KCNQ1 tend to affect the activation of the latter through different structural re-arrangements in the KCNQ1 channel. In our recent study [15] we employed advanced modelling and MD simulations, including the replica-exchange MD (REMD) approach, to build a comprehensive 3D structure of the KCNQ1/KCNE1 complex. In this study [15], we initially constructed a 3D structure of the human KCNQ1 channel, using the homology modelling approach with the X-ray crystal structure of a Kv1.2-Kv2.1 paddle chimera channel (PDB ID: 2R9R) as the template. REMD approach was implemented to refine the S1-S2 helices from our KCNQ1 model. Later, we performed ~800 ns long MD simulations of the independent structures of KCNQ1 model and the known X-ray crystal structure of human KCNE1 protein (PDB ID: 2K21). Cluster analyses were then performed on the MD trajectories and resulted in the extraction of low-energy dominant conformations of human KCNQ1 model and KCNE1. These dominant conformations were used to perform rigorous ensemble-based protein-protein docking in order to build comprehensive structural models of the KCNQ1/KCNE1 complex, which were finally refined through long-scale MD simulations [15]. The results from this study [15] revealed the dynamic behaviors of the KCNQ1 alone and the KCNQ1/KCNE1 complex and revealed that the protein-protein interactions have improved the structural stability of KCNQ1. This finding is consistent and complementary to the earlier studies [16–18]. In addition, in our previous classical MD simulations, we found that the ions could enter the open pore of the KCNQ1 channel (particularly in the absence of KCNE1 protein); however, they were not able to pass through the selectivity filter residues and therefore fell back into the intra cellular region. Further, we observed apparent differences in the time and frequency of ions permeating into the pore of the KCNQ1 channel in the presence and absence of KCNE1 protein. This indicated that the KCNQ1-KCNE1 interactions can affect ion permeation, however, we were not able to assess the molecular processes underpinning these differences using classical MD simulations. Until date, there have been limited studies that explicitly addressed the question of how the intermolecular interactions between KCNQ1 and KCNE1 impact the ion-permeation.

Therefore, in the current study, we aim at addressing this important question by using the structural models of the open-state human KCNQ1 structure and the human KCNQ1/KCNE1 complex that we developed in our earlier study [15]. In particular, we focus on understanding the structural and mechanistic details of the ion permeation through the open-state KCNQ1 structure and how these details change upon KCNQ1 interactions with KCNE1 and small molecule binding to the KCNQ1/KCNE1 complex. For this purpose, we employ steered molecular dynamics (SMD), an enhanced sampling approach, to estimate the magnitude of the force required to pull a potassium ion from the intra-cellular to the extracellular region and through the pores of the unbound-KCNQ1 channel and the KCNQ1/KCNE1 complex. This is useful to develop qualitative and quantitative insights into the ion permeation mechanisms in this ion channel complex[19–22]. SMD is a popular approach that has been previously applied to study a number of ion channels, including KvAP voltage-gated potassium channel, voltage-gated sodium ion channel, calcium ion channels, mechanosensitive channels, etc [22–26].

In addition, we have also extended our study to reveal the effects of small-molecule binding on the potassium ion permeation through the KCNQ1/KCNE1 complex. It is known that several drugs exhibit severe cardiotoxic effects due to binding into the KCNQ1/KCNE1 complex and blocking the permeation of potassium ion and thereby perturbing the generation of the I_Ks_ current [27–31]. Hence, it is also important to understand how blockers with different binding affinity towards KCNQ1, affect potassium ion permeation. To address this, we have performed molecular docking calculations of Chromanol 293B, a well-known KCNQ1 blocker and its derivatives [32,33], into the pore region of the KCNQ1/KCNE1 complex. Subsequently, SMD simulations were performed to pull the ion from the intracellular region to the extracellular environment, but in the presence of the bound-ligand. Our SMD simulations are able to discriminate between the high-affinity blockers and weak blockers, that are in agreement with the previous biochemistry results. Thus, the study presents comprehensive atomic-level details about the impacts of protein-protein interactions and ligand-binding on the K+ ion permeation through the KCNQ1 channel.

## Methods

### Classical MD simulations of the unbound systems

The structural models of the KCNQ1 protein and the KCNQ1/KCNE1 complex were embedded in membrane and were subjected to long MD simulations. The KCNQ1 and KCNQ1/KCNE1 complex, were obtained from homology modelling and protein-protein docking and simulations, as detailed in our previous paper [15]. The full membrane-bound systems were built using the Membrane Builder program of CHARMM GUI (http://www.charmmgui.org). The tetrameric protein (or protein-protein system) was embedded in a bilayer of Palmitoyloleoylphosphatidylcholine (POPC) and Phosphatidylinositol 4,5-bisphosphate (PIP_2_) in the ratio of 10:1, respectively. The system was further hydrated with 20 Å (TIP3P water model) on upper and lower leaflets. An ionic concentration of 150 mM KCl solution was maintained in the system, both in the upper and lower regions and neutralized with counter ions. Protein, lipids and ion parameters were assigned using the CHARMM36 force field. NAMD package [34], version 2.10 and 4,096 processors on the Blue Gene\Q supercomputer were employed for running the Molecular Dynamics (MD) simulations.

The solvated, membrane-bound systems underwent two stages of energy minimizations. In the first minimization round of 50,000 steps, the protein and the lipid heads were fixed; whereas the lipid tails, water and ions were allowed to relax. This step was essential to remove any existing steric clashes that might have risen from the improper packing of the membrane around the protein. During the second stage of minimization, a constraint of 100 kcal/mol was placed on the entire system and energy minimization was performed for 50,000 steps. This constraint was gradually removed during four more rounds of minimization. Each minimization stage was of 50,000 steps and the constraining forces were reduced to 50, 10, 5 and finally 1 kcal/mol. The energy minimized systems were then heated to 310 K for 1 ns, while retaining the 1 kcal/mol backbone restraints. Next, we performed NPT equilibration of the systems for 250 ps each. Finally, the production MD simulations of the systems, with an integration time step of 2 fs and periodic boundary conditions, were carried out for ~240 ns timescale. The Langevin thermostat was employed to maintain the constant temperature (310 K) and pressure (1 bar) during the production simulations. Bonded interactions were computed every time step, short-range non-bonded interactions every two time steps, and long-range electrostatic interactions every four time steps. A cutoff of 12 Å was used for van der Waals and short-range electrostatic interactions; with a switching function starting at 10 Å for van der Waals interactions to ensure a smooth cutoff. The simulations were performed under periodic boundary conditions, with full-system, long-range electrostatics calculated by using the particle-mesh Ewald (PME) method. The unit cells were large enough such that adjacent copies of the protein were never close enough for making short-range interactions.

### Clustering for dominant conformations of KCNQ1/KCNE1 complex

Later, the 240 ns long MD trajectory of KCNQ1/KCNE1 complex was clustered to identify the most dominant conformations of the complex to perform small-molecular docking. For this purpose, we adopted the Average-Linkage algorithm using a code in PTRAJ program of AMBER [35]. We ran the average-linkage algorithm for a number of clusters ranging from 5 to 100. Structures were extracted at 4 ps intervals over the entire simulation time (240 ns). In this algorithm, cluster-to-cluster distance is defined as the average of all distances between individual points of the two clusters. Clustering quality is determined through the calculation of a number of clustering metrics including the Davies-Bouldin index (DBI) [36] and the "elbow criterion" [37]. These metrics help in identifying the optimal number of clusters to be extracted and their population size. In order to remove the extra noise from the data as a result of rotations and translations, all the non-hydrogen heavy atoms were fitted to the minimized initial structure. Next, the RMSD is used to cluster the residues at the binding site of the ligands. These residues were clustered into groups of similar conformations using the atom-positional RMSD, as the similarity criterion. In each cluster, the structure that has the minimum RMSD (also called the cluster centroid) was chosen as the cluster representative. 15 dominant conformations were obtained from the clustering analyses and alignment of the DBI and SSR/SST parameters to be used in the subsequent docking simulations. These 15 conformations represent more than 95% of the structure variability during the MD trajectory.

### Small-molecule docking

Molecular docking calculations were carried out using the most recent version of smina [38]. Smina is a version of AutoDock Vina which offers a better control over the docking and scoring parameters [39]. The protein structures were prepared using the protein preparation wizard in the Schrodinger software package [40]. The protonation states were assigned at the pH of 7. Protein structures were then saved as PDB files and converted to PDBQT format using the AutoDock Tools [41] to be used as inputs for smina.

Ligand structures were prepared using the ligprep [42] module of Schrodinger and saved as mol2 files. The ligand protonation states and tautomeric states were assigned at neutral pH. The geometry of the ligands were optimized through the OPLS2005 force field [43]. The docking search space was confined to a 20*20*20 Å box around the ligand-binding site with an exhaustiveness search parameter of 20 (default is 8). The binding site residues of Chromanol 293B were obtained from a mutational study by Lerche et al. [44] who had confirmed the residues responsible for interaction with Chromanol 293 B. These residues included Thr312, Phe340 and Ile337 from the four subunits. The ligand-bound KCNQ1/KCNE1 complexes, obtained from the docking calculations, were also embedded in a mixed POPC/PIP_2_ membrane bi-layer and subjected to ~4 ns long MD simulations. The preparation and MD simulations of the ligand-bound systems were carried out exactly in the same sequence of processes performed for the unbound systems, as detailed above.

### Steered molecular dynamics simulation

The starting structures of KCNQ1, ligand-unbound KCNQ1/KCNE1 and the ligand-bound KCNQ1/KCNE1 complex for the SMD simulations were obtained from their respective classical MD trajectories. The parameters for the SMD simulations were mostly same as the classical MD, except for the application of an external force to pull a potassium ion from the intracellular environment to the extracellular bulk region. The force was applied along a vector normal to the x-axis pointing from the axis to the initial position of the atom. During the simulation, C*α* atoms of the Asp301 residue located on the S5/P-loop linker in the four subunits were constrained along the Z-direction with a force of 1 kcal/mol. This was done to prevent any structural drifts in the protein and its location in the membrane, while the ion was being pulled. Each SMD simulations were carried out for 4 ns long time scale using a spring constant of 4 kcal/mol/Å and a constant velocity of 0.025Å/ps. The protein experienced no appreciable drift in the plane of the membrane, so the applied forces may be considered to be radial at all points in the simulations. Repeated SMD simulations were performed for the different systems to ensure reproducibility of the results (refer to supplementary information, S1–S3 Figs). The RMSD graphs for the SMD simulations are also provided in the supplementary information (See S4 Fig), which confirms that the application of external force did not make any significant impacts on the overall structures of the systems. All the SMD simulations in this work were performed using NAMD 2.9 [34] package.

### Analysis & visualization

VMD [45] and Chimera suite [46] were employed for visualization and analyses of MD trajectories in this work. Pore radius profiles were calculated using HOLE program [47,48]. All the plots discussed in this work were generated using Gnuplot and GraphPad Prism version 6.0 [GraphPad Software, La Jolla California USA, www.graphpad.com].

## Results and discussion

### Classical MD simulations

**Fig 1:**
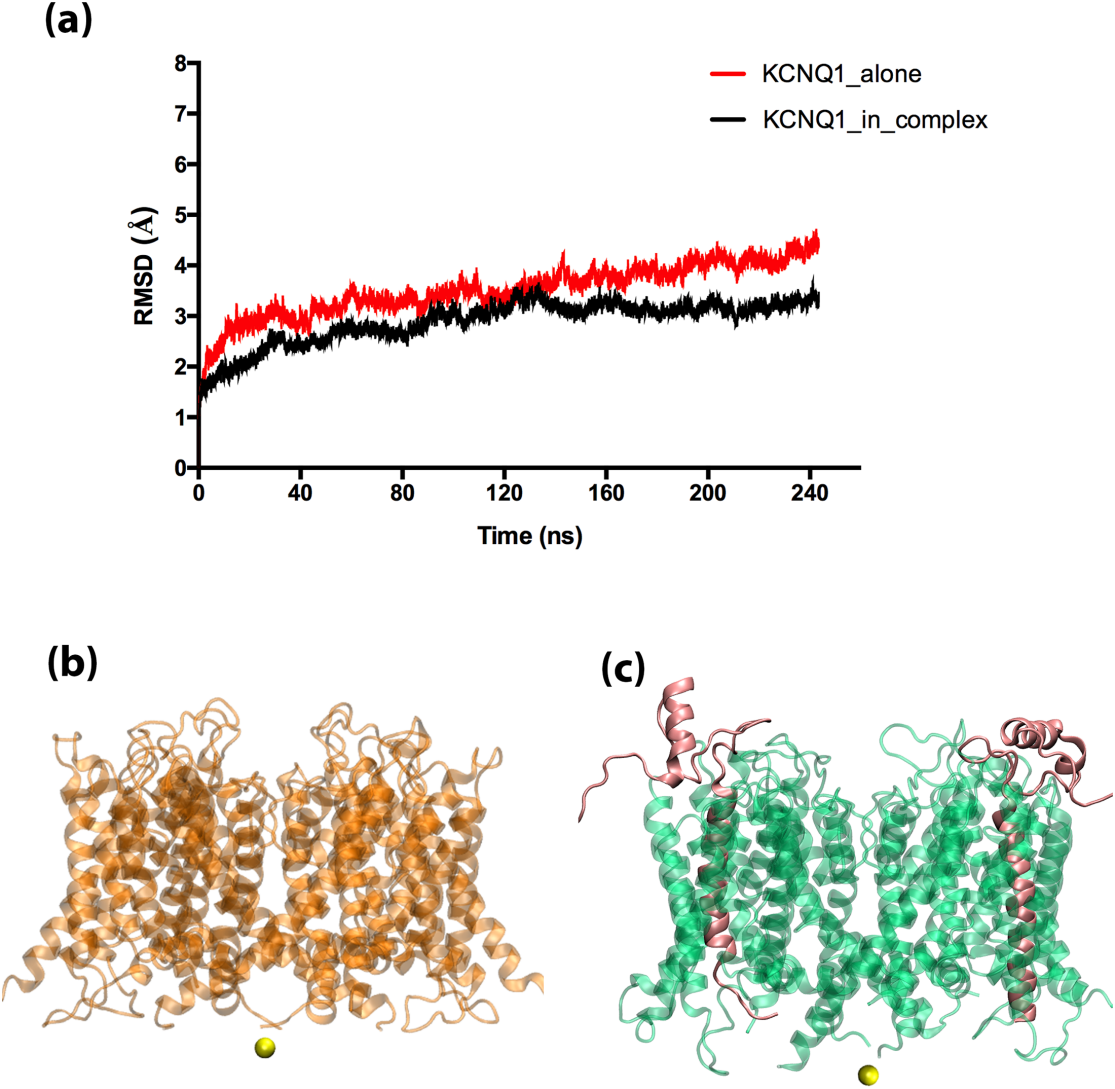
The starting systems for SMD simulation. (a) RMSD plots of classical MD simulation for KCNQ1 and KCNQ1/KCNE1 Systems, (b) structure of KCNQ1 protein alone, (c) structure of KCNQ1/KCNE1 protein complex.

The KCNQ1 and KCNQ1/KCNE1 systems were thoroughly equilibrated during ~240 ns long classical MD simulations. We expect that the systems are stabilized during MD equilibration and could offer insights about their relevant dynamic changes under the influence of surrounding environment (water, lipids, ions). The Fig 1a presents the root mean square deviation (RMSD) of the protein backbone in KCNQ1 and KCNQ1/KCNE1 complex during the MD simulations. As it can be seen in the Fig 1a, there were only ≤ 2 Å deviation in the RMSD values that indicates the overall stability of the systems during MD simulations. In particular, it can be seen that the changes in the RMSD values for the KCNQ1/KCNE1 complex were slightly lesser than those seen in the un-complexed KCNQ1 protein. This describes that the intermolecular protein-protein interactions have added some degree of rigidity to the complex. The only flexible regions during our analyses (of the MD trajectories) were related to the loop regions and linkers in between the segments, which is an expected phenomenon. The more specific details about the model and the dynamic changes of the systems during MD simulations are reported in detail in our earlier work [15]. But one important observation we made was that, despite the model being an open state, the potassium ions freely entered into the pore; however, they were not able to cross through the selectivity filter regions in the ion channel. As a result, we noticed that the ions either stayed inside the pore or moved back into the intracellular environment. Understandably, this is a known limitation of classical MD simulations. The potassium ion permeation through the selectivity filter barriers of the KCNQ1 channel would require several nanoseconds to microseconds of MD simulations, which is not always practical. Particularly, in this study, we compare an un-complexed KCNQ1 and a KCNQ1/KCNE1 complex, and it is nearly impossible to study the impacts of protein-protein interaction on the ion permeation mechanisms only based on extensively long classical MD trajectories. As a result, we decided to dedicate this work for employing alternative enhanced sampling SMD approach to gain mechanistic insights into ion permeation processes in the un-complexed KCNQ1structure and how they are affected by intermolecular KCNQ1/KCNE1 interactions. For the purpose, we sampled the MD trajectories and selected the snapshots for KCNQ1 and KCNQ1/KCNE1 complex, in which a potassium ion is relatively placed in the same position near the intracellular entrance of the channel (see in Fig 1b and 1c). This reduces any possible bias that may arise from manually placing an ion at the position(s) of interest within the system(s).

### Steered molecular dynamics (SMD) simulations

SMD simulations were initially performed for the two systems: (1) the KCNQ1 protein; and (2) the KCNQ1/KCNE1 complex. During the SMD simulations, a single K^+^ ion was pulled from the intracellular region (at the bottom of the pore), through the KCNQ1 pore, to the extracellular region. The length of this pathway was ~34 Å long (i.e. starting from the intracellular channel entrance to the extracellular loops above the selectivity filter). Each of the SMD simulation was carried out for 4 ns and with a combination of 4 kcal/mol/Å of spring constant and 0.025 Å/ps of velocity. The choice of the force and velocity parameters was based on a previous study from our group, in which we benchmarked the different forces and velocities to find the optimum combination for ion channels [22]. Our findings from these SMD simulations for the ligand-free systems are presented in the following sections.

#### Ion permeation in KCNQ1 protein

**Fig 2:**
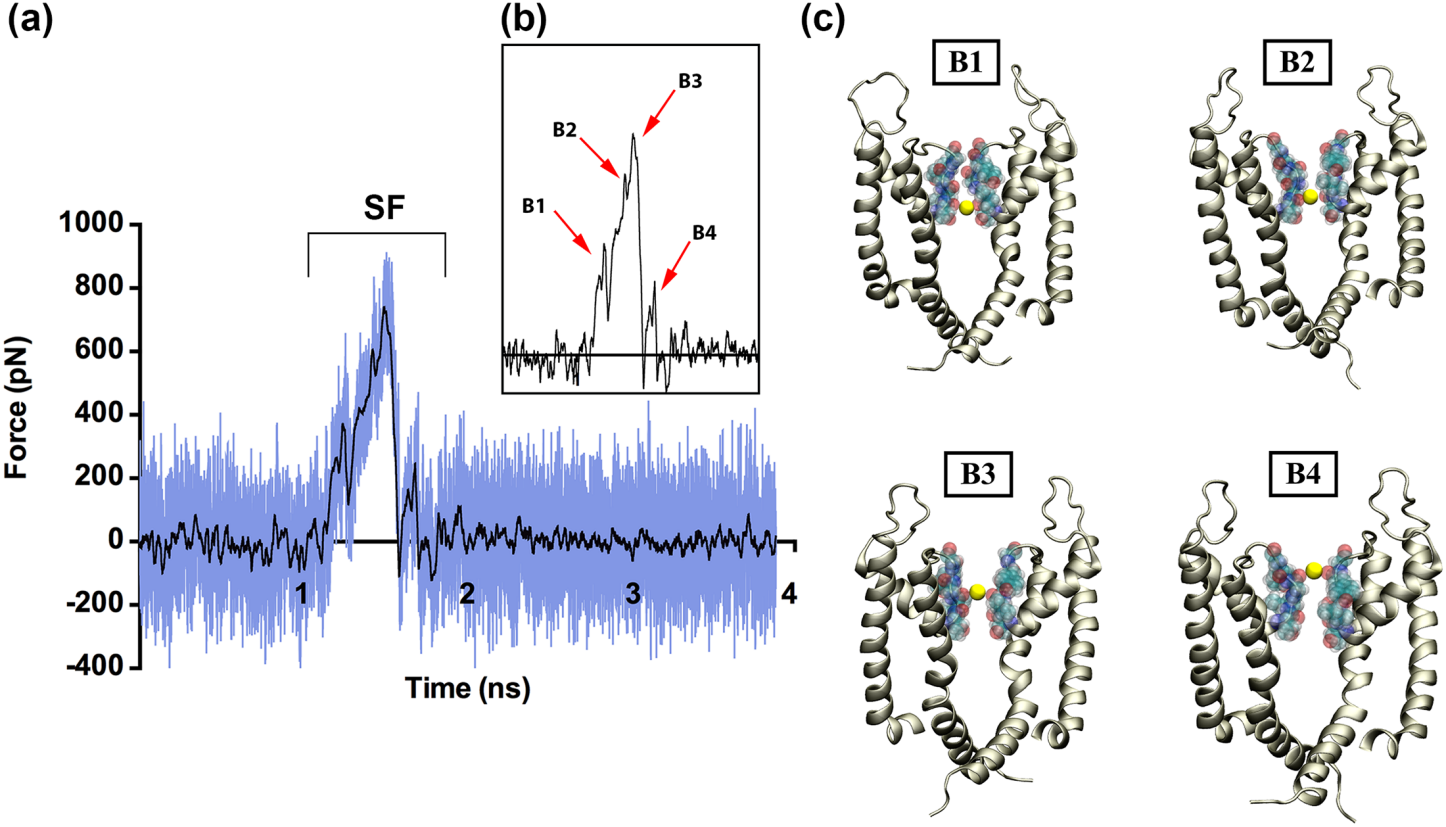
The SMD force profile of K^+^ ion pulled through the KCNQ1 protein alone. (a) The force profile for the potassium ion pulled through eh KCNQ1 channel pore, showing the high peak corresponding to the selectivity filter (SF) of the protein. (b) The zoomed-in peaks of the force profile corresponding to the energy barriers marked as B1, B2, B3 and B4. (c) Snapshots from the SMD showing the location of the ion at the different binding sites (B1, B2, B3 and B4) in the selectivity filter with respect to the force profiles. The potassium ion is shown in yellow, the S5, S6 and P-loop of two subunits are shown in cartoon. The KCNQ1 VSD and the other two subunits are not shown for clarity.

Fig 2a presents the SMD force profile for pulling a potassium ion through the pore of the un-complexed KCNQ1 channel. In a standard SMD profile graph, hills represent different potential energy barriers along the ion permeation pathways, thus, necessitating larger pulling force to release the ion from the respective sites. The plateaus appear whenever the ion easily escapes without any potential barriers along the path. In case of the lone KCNQ1 protein, until ~1 ns of the SMD simulation, the force profile is flat, indicating the absence of any barricades in its permeation path. However, at ~1.2 ns, the ion encounters the selectivity filter residues that cause significant energetic barriers in further ion permeation. The region corresponding to the selectivity filter is marked as ‘SF’ on the plot (Fig 2a). Thus, the force required for pulling the ion from the impact of selectivity filter residues required as much as ~600 pN force in the SMD simulations. Accordingly, the force plot for the SMD simulation of KCNQ1 (shown in Fig 2b) shows peaks of different intensities within ~1–2 ns time scale. We name these peaks as B1-B4 in this study (see Fig 2b). These peaks possibly indicate the presence of different binding sites for potassium ion at the selectivity filter region of the KCNQ1 channel, as shown in Fig 2c. It can be noted that B1 corresponds to the site where the potassium ion is coordinated with the strong electronegative carbonyl oxygen atoms of the four threonine residues (Thr312) from all the subunits. When the SMD external force reaches ~ 400 pN, the ion is released from the B1 site. Once released, it encounters the next two sites (B2 and B3), where the ion is obstructed by the strong electrostatic interactions rendered by Ile313 and Gly314 from all the four subunits. Hence, releasing the ion from the B2 and B3 sites required more external force of ~600 pN during the SMD simulations. It was noted (during the SMD simulations) that the Thr312 residues were involved in sliding the potassium ion from B1 to B2 site; whereas, the same role was played by Gly314 when the ion migrated from B3 to the B4 site. The final energetic barrier encounter by the ion in its’ permeation pathway was at site B4, where the ion was slowed down by the carbonyl oxygen atoms from two residues, Tyr315 and Gly316. To escape from this site, a small force of ~200 pN was required during the SMD simulations. It is clear from the plot that after being released from the B4 site at ~1.9 ns, the ion entered the extracellular region (or bulk environment) and thus, the SMD force profile remained flat until the end of ~4 ns long simulation.

Furthermore, we analyzed the presence and movement of water molecules during the SMD simulation. As shown in S5 Fig, the entrance pore is widely open for water molecules. However, as they approach the selectivity filter, the number of water molecules that can be accommodated is significantly reduced. This explains why the selectivity filter acts as a sieve and does not allow the passage of other ions of different size, density and charge to pass the pore. These findings are consistent with previous studies from the literature, related to the selectivity filter properties and the binding sites of potassium through the channel [49–52]. Our SMD simulations clearly identified four binding sites for potassium formed by the selectivity filter motif TIGYG (Thr-Ile-Gly-Tyr-Gly). We were also able to quantify their energy barriers along the pathway of the ion. For an ion to successfully pass through the filter, it has to overcome each of the carbonyl atom cages created by these residues, one after the other. The force needed for a potassium ion to migrate from the first binding site formed by Thr residues (B1) is almost 50% lesser than the force for pulling ion from the binding sites formed by the Ile and Gly residues (B2 and B3). The characteristics of the selectivity filter in voltage-gated potassium ion channels are well studied [51,53–55]. In KCNQ1, there are four well-defined binding sites formed by the carbonyl atoms of the TIGYG residues that capture the ion and allow its sequential passage through the selectivity filter [56]. Our SMD simulations showed that the 2^nd^ and 3^rd^ binding sites i.e., B2 and B3 serve as the rate-limiting sites within the KCNQ1 channel.

#### Ion permeation in KCNQ1/KCNE1 complex

**Fig 3:**
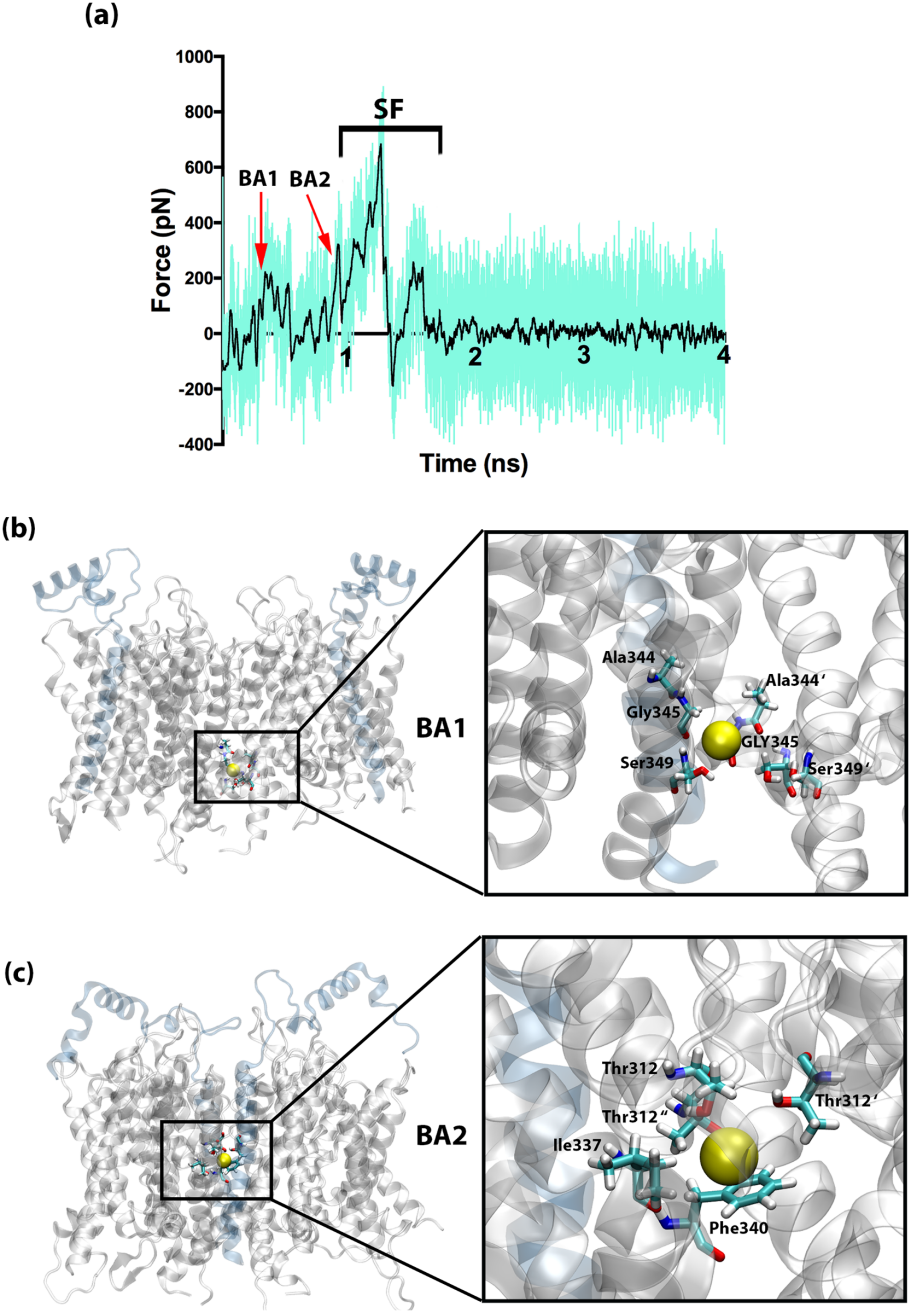
The force profile of K^+^ ion pulled through the KCNQ1/KCNE1 protein complex. (a) The SMD force profile to show the different peaks formed by the barriers marked as BA1, BA2 and SF (selectivity filter), (b) Close up view of the BA1 barrier, (c) Close up view of the BA2 barrier. The potassium ion is shown in yellow sphere, the protein structure is shown in cartoon presentation and the residues are depicted by bonds.

To understand the effects of KCNE1 on the ion permeation process, we performed the SMD simulations (with the same parameters described above) on the KCNQ1/KCNE1 complex. The results of the SMD simulations are shown in Fig 3a. When compared to the SMD force profile for ion permeation in KCNQ1 protein (in Fig 2a), the force profile for the protein-protein complexes (in Fig 3a) exhibit some additional peaks. Particularly, these new peaks (marked as BA1 and BA2 in Fig 3a) are seen during the initial stage of the SMD simulations. This clearly indicates the presence of some energetic barriers (not seen in un-complexed structure of KCNQ1) that are imposed upon the interactions of KCNQ1 with the KCNE1 protein. The two peaks, named as BA1 and BA2 in this study, are seen at ~0.5 ns and 1 ns of SMD simulations. While the first peak (i.e., BA1) is clearly independent of the others, the second peak (BA2) is seen as a shoulder of the main ‘SF’ peak, which is also found in the un-complexed structure. Close inspection of the SMD trajectories revealed that the BA1 site is located just below the S6 helices (See Fig 3b). When the potassium ion reached this site, it was encircled and trapped by electrostatic interactions rendered by different residues, such as Ser349, Ala344, Gly345 from different subunits, thus requiring a force of ~200 pN to permeate further into the pore. Single point mutations at these sites in KCNQ1 have been associated with LQT1. For example, a natural variant of Ser349Trp mutation has been found in LQTS1 patients, however, the reason behind the effect of this mutation on the disease pathogenicity is still not clear [57]. Our SMD simulations clearly state that this residue (i.e., Ser349) is located at the entrance of the pore region and results in an energy barrier for the ion passage. In this context, it is understandable that mutation of this serine residue with a bulkier tryptophan residue would cause more strong constriction at the pore, thereby, leading to the channel loss-of-function. Similarly, mutation of Ala344 to valine (i.e., Ala344Val) has been reported in LQT1 patients [58]. This mutation has been categorized as a dominant-negative mutation[59], meaning that it acts antagonistically to that of the wild-type gene. The Gly345Arg mutation has been associated with familial sudden death in LQT1 patients [59]. This indicates that the BA1 site (formed by Ser349, Ala344, Gly345) plays a very important role in the functionality of the KCNQ1 ion channel. After releasing the ion from this site, with the help of an SMD external force, the ion encounters its next energetic barrier, located close to the selectivity filter at the pore region of the channel marked as BA2 and shown in Fig 3c. At the BA2 site, a different set of residues (composed of Thr312 from all four subunits, Ile337 and Phe340) from the S6 helix and S5-P-loop linker obstructs the ion movement. In this path, the cation-π interaction between the pulled K^+^ ion and Phe340 seems to form a major barrier that requires ~280 pN to break-free the ion. A previous study employed alanine-scanning single residue mutagenesis experiments found that Thr312, Ile337 and Phe340 are among the most significant molecular determinants of KCNQ1 blockade [60]. This confirmed that Phe340 played a very important role in the interactions with the KCNE1 channel. Mutations at this site using either an alanine residue or a tryptophan residue led to the complete loss of functional modulation of KCNQ1 by KCNE1 [61]. Since, our SMD simulations clearly show that the KCNE1 interactions constricts the KCNQ1 pore, it could be hypothesized that Phe340Ala or Phe340Trp mutation can affect the closing of the channel. This proposal is supported by an earlier finding [7] that a mutation at Phe340 site could disrupt the close state of KCNQ1 and can modify its inactivation, which could be linked with LQTS [7]. A comprehensive list of all known LQTS1-linked single point mutations and their reported properties are provided in S1 Table.

**Fig 4:**
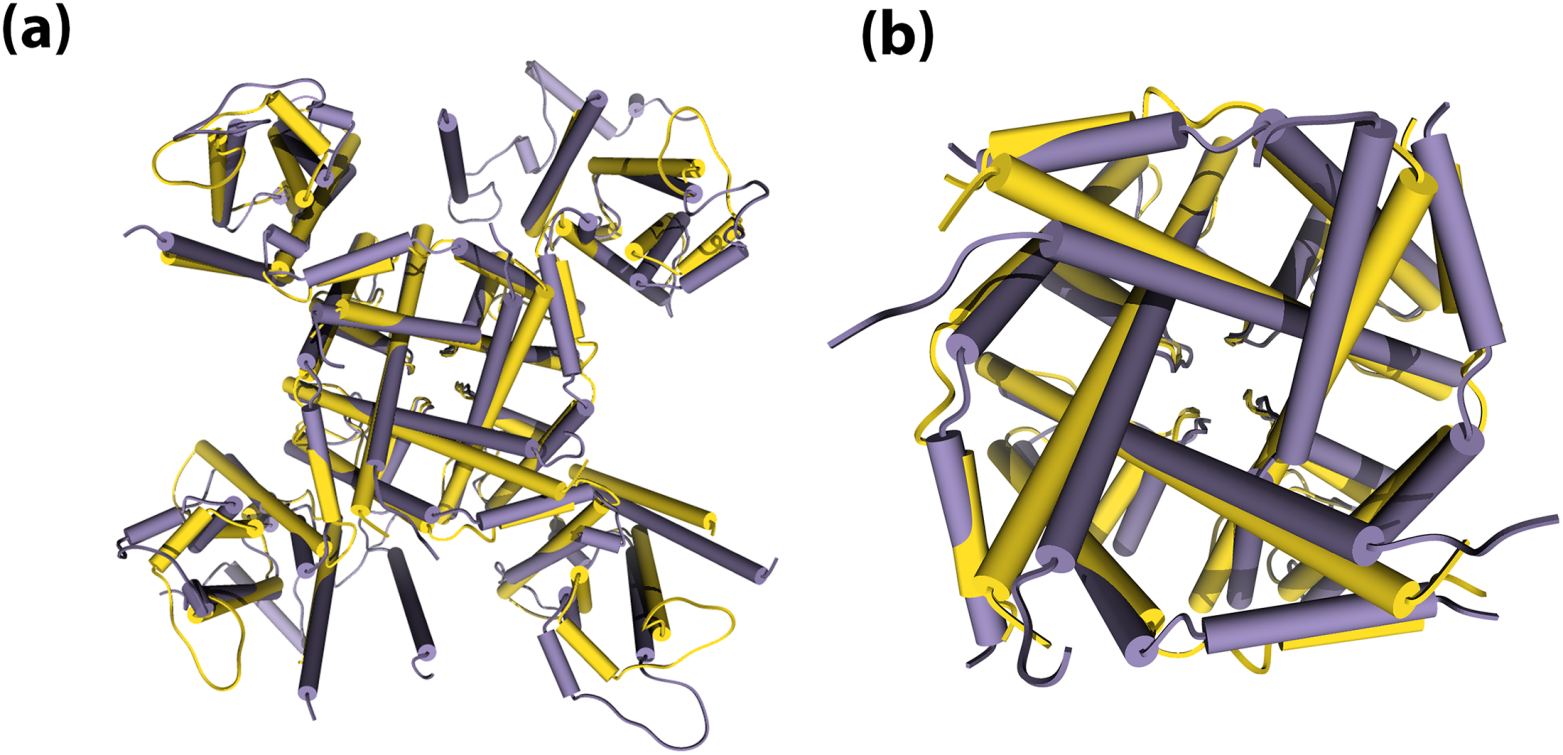
Superimposition of the KCNQ1 alone (yellow) and KCNQ1/KCNE1 complex (purple). (a) rear view of the channel, (b) zoomed view to show the shift in the S6 helices.

It is important to highlight that the two sites (BA1 and BA2) and the corresponding force peaks were not seen in the SMD simulations of KCNQ1 protein. This describes the possible impact of KCNQ1/KCNE1 interactions on the pore domain of the KCNQ1 channel. Fig 4 present the superimposed 3D structures of the un-complexed KCNQ1 (in yellow) and the KCNQ1/KCNE1 complex (in purple). It can be observed that there is a clear shrink in the pore-opening (in Fig 4b) that is possibly caused by the inward-shift of the S6 helices leading to the iris-like change in the pore opening. This finding is useful to explain the previous experimental reports that identified dramatic slowdown in the KCNQ1 activation due to its’ interactions with KCNE1 protein [7,62,63]. Further, a previous study by Hoshi et al. [64] also hypothesized that the elimination of the slow inactivation in potassium channels, might be linked to structural rearrangement in the pore and/or constriction of the selectivity filter region. In line with these earlier hypotheses, our SMD simulations provide in-depth qualitative and quantitative insights into the effects of KCNE1 interactions on the structural rearrangements in KCNQ1 and, thus, in the ion permeation process as well. It is important to note that, the KCNE1 interactions, however, do not cause any significant changes to the selectivity filter residues of KCNQ1, as suggested by Xu et al [14]. This is explained by our SMD simulations (as shown in Fig 3a). After releasing from the BA2 site in the KCNQ1/KCNE1 complex, the ion entered into the selectivity filter segment and it can be seen that the barriers and the corresponding peaks in this SMD force profile (Fig 3a) are all almost similar to those seen in the un-complexed KCNQ1 simulation (Fig 2b). That is, four peaks related to B1-B4 sites, which are collectively marked as SF, are all seen in the force profile of KCNQ1/KCNE1 after ~1 ns SMD simulations. This confirms that the interactions of KCNE1 do not induce any significant structural changes in the selectivity filter of KCNQ1 channel and its main influence is manifested at the pore region.

#### Pore dimension analysis of lone KCNQ1 and KCNQ1/KCNE1 complex

We also analysed the pore dimensions of the KCNQ1 ion channel and the KCNQ1/KCNE1 complex, using the HOLE program [47,48]. The HOLE program adopts a Monte Carlo simulated annealing approach to find the best path for a sphere (of variable radius) to squeeze through the channel. HOLE has been successfully used in complementing the analysis of ion channels in several studies [65–68]. The main objective of this analysis was to support our findings related to the identified force profiles of K^+^ ion in the two systems; i.e. KCNQ1 alone, and KCNQ1 in complex with KCNE1.

**Fig 5:**
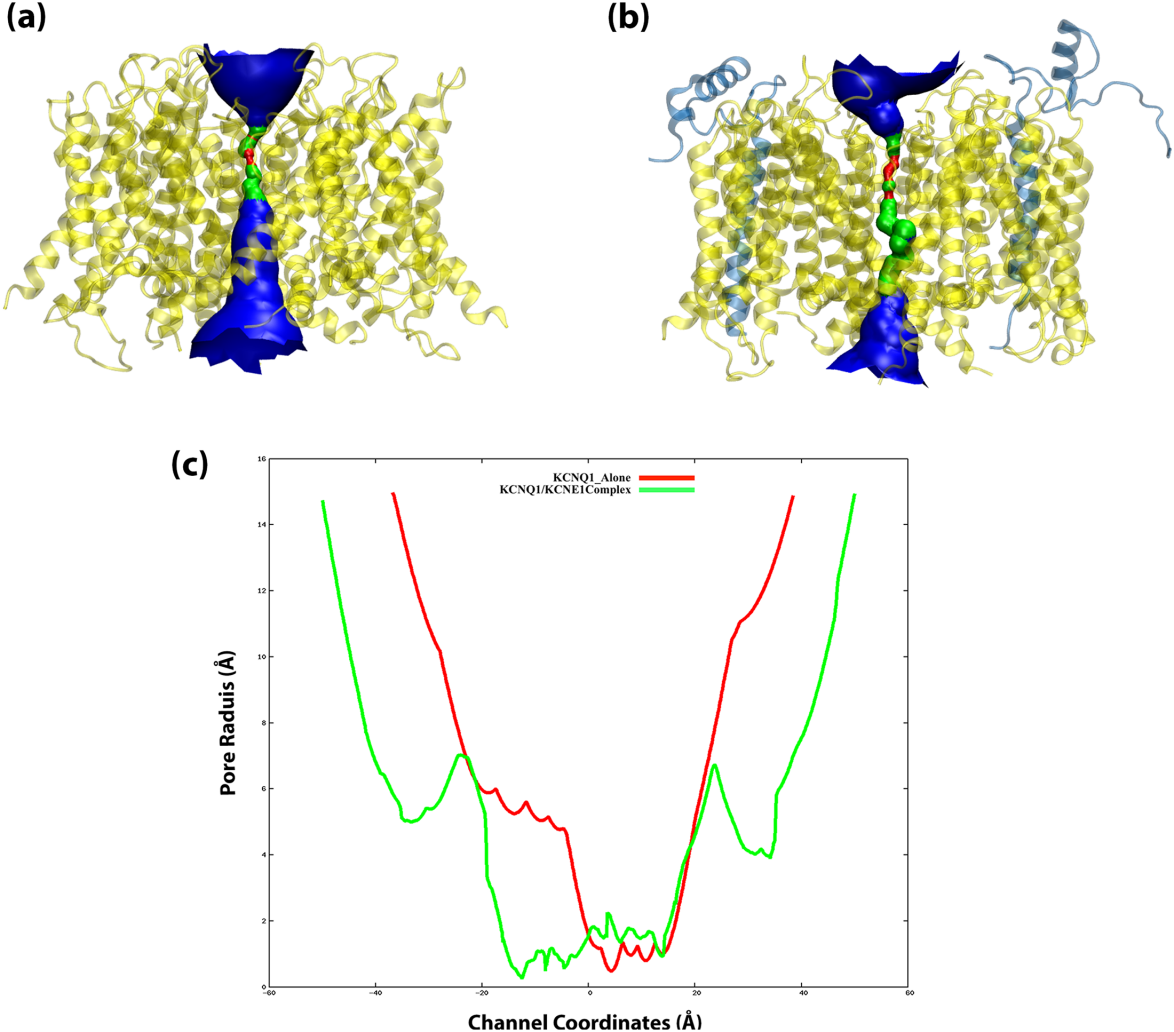
The dimensions of the pore shown in surface representation. For the two model states: (a) KCNQ1 without KCNE1, (b) KCNQ1 in complex with KCNE1. *Colour code: Red is where the pore radius is too tight for a water molecule. Green where there is room for a single water molecule. Blue is where the radius is double the minimum for a single water molecule. (c) Pore radius plot of the KCNQ1 alone (red) and KCNQ1/KCNE1 complex (green) systems. There is a continuous constriction from the pore opening up to the selectivity filter in the KCNQ1/KCNE1 system as compared to the KCNQ1 pore which has a wide opening throughout the pore.

As shown in Fig 5a, the pore dimensions in the KCNQ1 protein (without KCNE1 interactions) have a wide opening in the bottom that continues until the selectivity filter region. This indicates that the channel has a completely open and wide pore without any constriction along the ion permeation pathway. The narrowing of the pore begins only at the selectivity filter region. This topology was found to remain stable throughout the long-scale molecular dynamics simulations. See S6 Fig for the comparison of the pore topology of structures from the beginning of the simulations vs. after the classical MD simulation. This confirms that the pore constriction did not occur as a result of an artefact from our MD simulations. Nevertheless, the pore dimensions in the KCNQ1/KCNE1 complex (shown in Fig 5b) has significantly contracted near the bottom-opening. This represents the site BA1 identified in our SMD simulations. Further, the pore becomes even narrower at the proximity of the selectivity filter (shown in green color), which is the site BA2 in our SMD simulations (Fig 3c). We also analyzed the radius of the pore in the two systems (as shown in Fig 5c). The red color line shows the radius of the pore domain in the KCNQ1 system. The pore opening has a radius of around 6 Å, which reduces to less than 2 Å, while approaching the selectivity filter. However, the radius of the pore in the KCNQ1/KCNE1 system (green color) shows a completely different pattern. The reduction in the radius of the pore begins early on at the opening of the pore domain, below the selectivity filter. The radius at this region is around 4 Å, which increases to ~7 Å marking the pore cavity. The radius of the pore is again reduced to ~0.4 Å and continues to be narrow along the selectivity filter. Therefore, it is apparent that the protein-protein interactions between KCNE1 and KCNQ1 have led to significant constriction in the pore segment of the latter. And as a result, extra small energetic barriers are seen for the KCNQ1/KCNE1 complex, which are rightly captured by our SMD simulations.

### Binding of small-molecules onto the KCNQ1/KCNE1 complex

**Fig 6:**
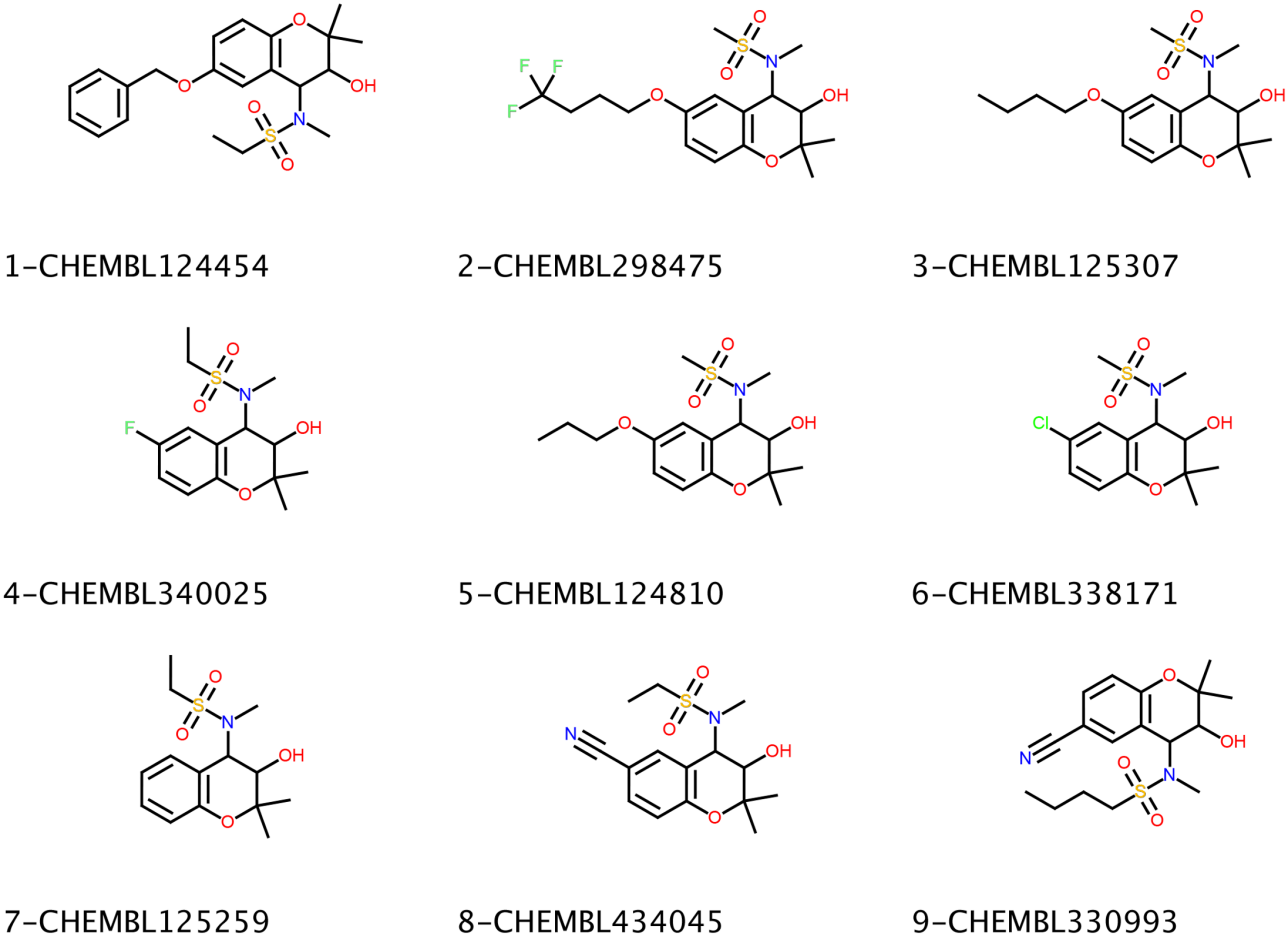
The 2D structures of the Chromanol 293B and its derivatives employed in the molecular docking calculations. The 2D structures of the compounds and their respective ChEMBL identification numbers are provided.

In order to study the effects of small-molecule binding on the ion permeation, we initially filtered a panel of known channel blockers of the I_KS_ current from the ChEMBL database [69]. For this purpose, Chromanol 293B and its derivatives that showed different range of binding affinity (IC_50_: 50 nM to 58,000 nM) towards the KCNQ1 channel, as determined by experiments, were selected. Chromanol 293B was discovered in 1996 [32,70,71] as a selective blocker of the I_KS_ current. Furthermore, Chromanol 293B was also found exhibit affinity for the open state of the KCNQ1 channel [44] and was therefore the best choice for our model, which is also built in its open state. In 2001, Gerlach et al. [33] synthesized several derivatives of this lead compound (Chromanol 293B) which in addition to Chromanol 293B are included our study. Out of the different stereoisomers of the compounds we selected the most active variants to test on the KCNQ1/KCNE1 system. The structures and CHEMBL_ID of these compounds are documented in Fig 6.

**Fig 7:**
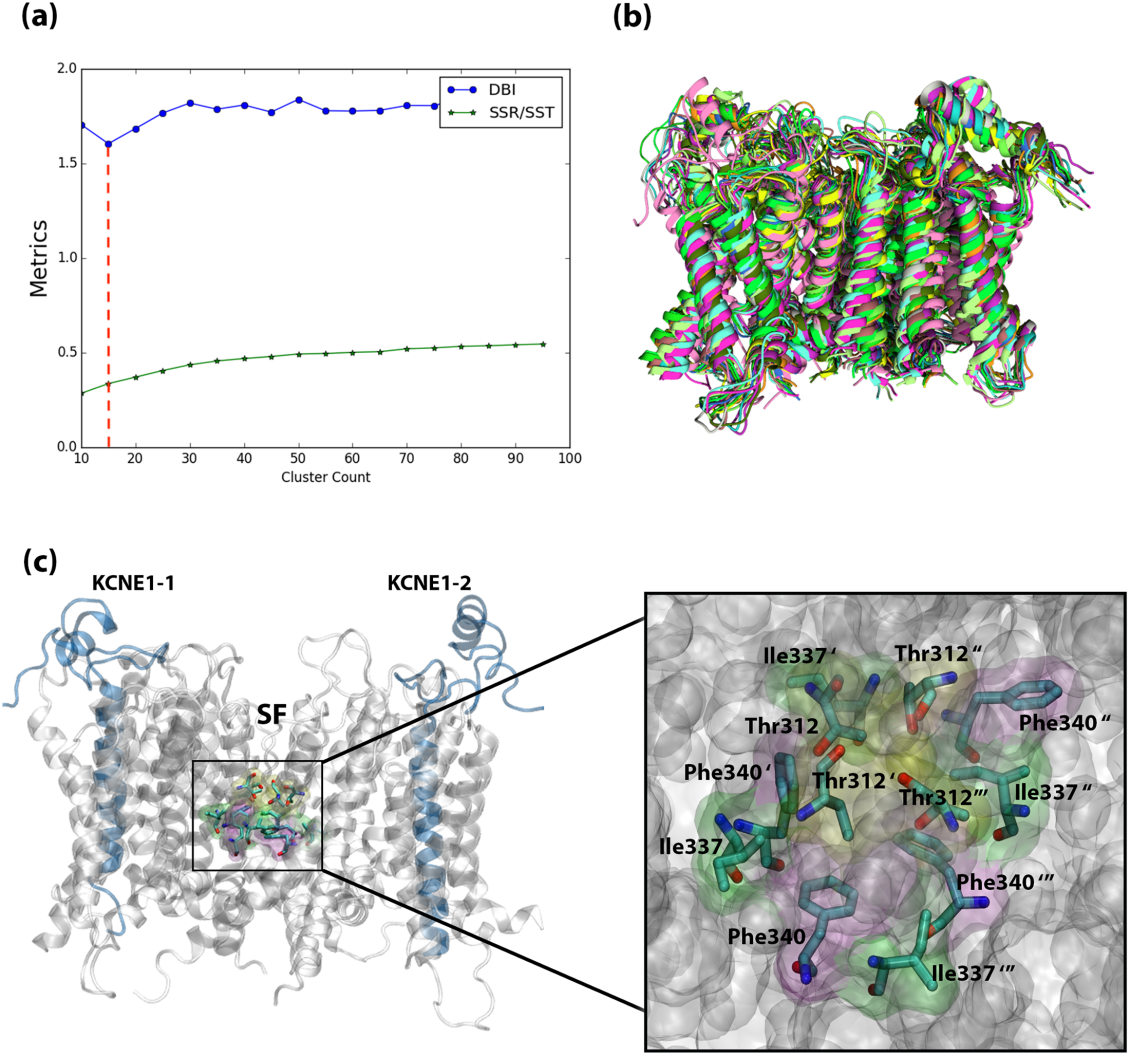
Clustering analysis of the KCNQ1/KCNE1 channel complex from the MD simulation based on the ligand binding site residues. (a) The clustering plot of DBI and SSR/SST parameters, (b) The 15 cluster representative conformations of the KCNQ1/KCNE1 complex protein. (c) The binding site residues of the Chromanol 293B and its derivatives shown in the structure of KCNQ1 (grey color cartoon) in complex with KCNE1 proteins (blue color cartoon). The binding cavity shown with bonds in licorice presentation, is located right below the selectivity filter (SF) of the channel. The residues with their names are shown in the close-up of the binding site.

In order to perform molecular docking, we initially performed RMSD-based clustering of our MD trajectory for KCNQ1/KCNE1 complex (Fig 7a) to extract dominant protein conformations (Fig 7b). The small molecule-binding site in the KCNQ1 channel (of the complex) was previously confirmed to be in the vicinity of Phe340 (binding site residues are shown in Fig 7c) [44]. As discussed in our previous studies [15,22,72–75], the optimum number of dominant protein conformations was obtained when the DBI reaches a local minimum with a flat SSR/SST line (elbow criterion). SSR is the sum of squared residual; the SST is the total sum of squares. For more details regarding the underlying theory of this clustering method, readers are encouraged to consult a review by Shao et al [37]. As can be seen in Fig 7a, clustering converges at approximately 15 protein conformations. These 15 conformations of the KCNQ1/KCNE1 complex (superimposed and given in Fig 7b) were later employed for our docking calculations. Subsequently, we docked the selected small-molecule ligands in this study, chromanol 293B and its derivatives in Fig 6, into all the 15 target structures. We have a defined binding site in the pore region of the KCNQ1 channel (shown in Fig 7c), to which the ligands were bound during docking calculations. Thus, a total of 135 independent docking calculation were performed. Molecular docking of each of the small molecules to an ensemble of protein conformations ensures the accommodation of the protein flexibility during the docking workflow in this study. This is important to address any conformational dynamics that can lead to a better docking pose for the tested compounds within the binding site of the protein. All docking simulations were performed using the smina docking tool [38], a version of AutoDock Vina which offers a better control over the docking and scoring parameters [39]. See the Methods section for parameters and details of the docking calculations.

#### Analysis of the protein-ligand binding modes

For each of the tested compounds, 20 different poses were obtained and ranked by AutoDock Vina scoring function [39]. The best poses for all the ligands determined by Vina were then re-ranked with two other machine learning scoring functions; the NNScore 2.0 [76] and the RF-Score-VS [77]. NNScore 2.0 is a neural-network based scoring function, devised to aid the computational identification of small-molecule ligands by providing a single p*K*_*d*_ (binding affinity) value. The RF-Score-VS [77], is another machine learning scoring function which has shown significant improvement in the performance of virtual screening studies [77]. Machine-learning scoring functions trained on protein-ligand complexes have shown great promise in small tailored studies as compared to conventional scoring functions such as Vina scoring function [77–79].

The final docking score considered for each ligand was the average of the results from three scoring functions, explained above, i.e. AutoDock Vina, NNScore 2.0 and RF-Score-VS. The poses were then closely visualized for their proper filling of the designated binding site (see Fig 7c). The binding site of the ligand was selected based on the study by Lerche et al. [44] who had investigated the binding mode of Chromanol 293B within the KCNQ1 pore, using a single point mutational approach. The binding site is located right below the selectivity filter and is formed by three residues from each subunit namely “Thr312, Ile337 and Phe340”. In the same study, it was found that the single point mutations at these specific positions intensely reduced current inhibition and had the strongest effects on blocking activity of Chromanol 293B [44].

**Fig 8:**
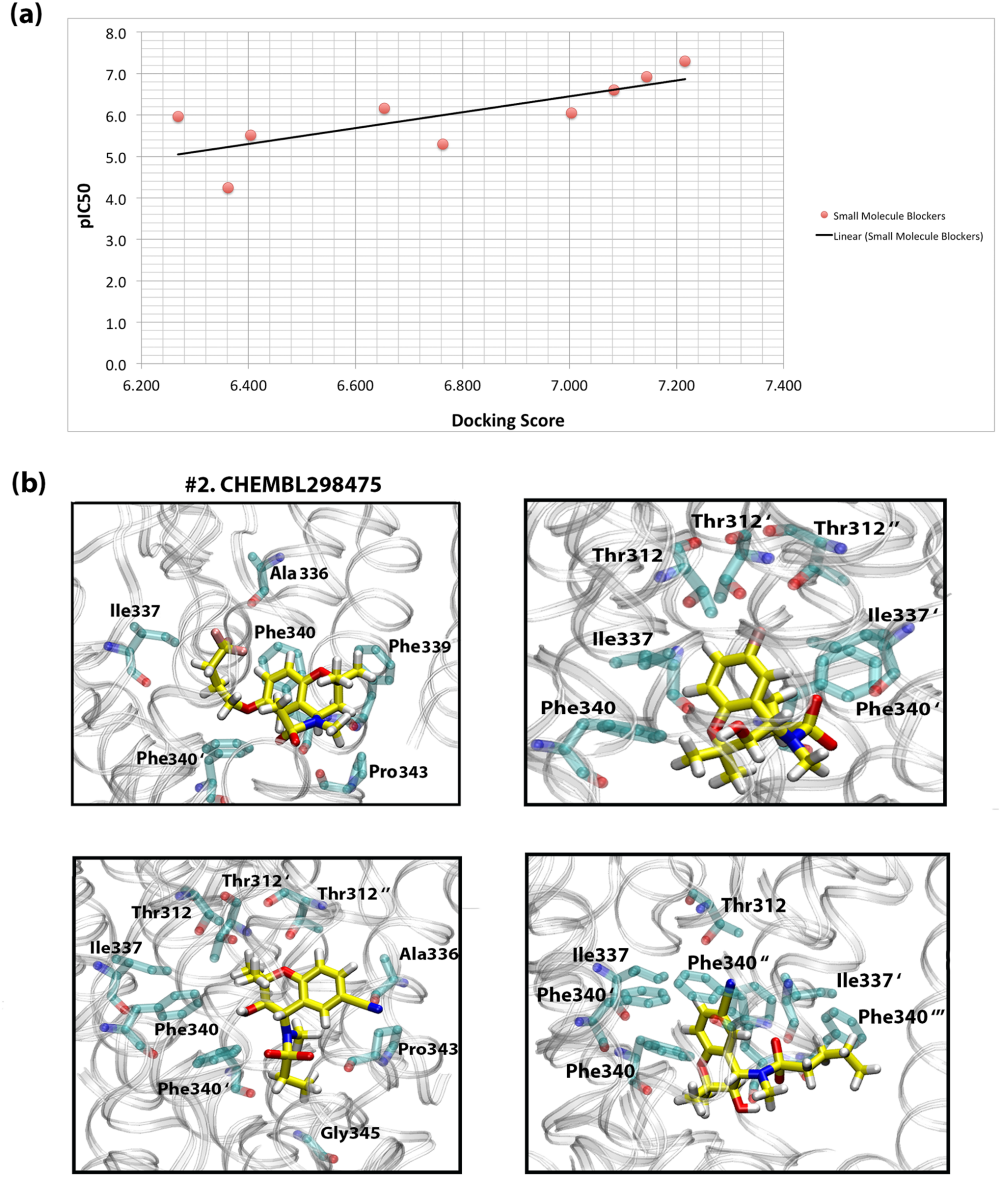
**(a) A 2D scatter plot of the compounds’ docking score vs. pIC**_**50**_**.** The linear line shows the positive correlation between the two variables (r_pearson_ = 0.7). **(b) The binding mode of ligands (#2, #4, #8 and #9) within the binding pocket of the channel.**

The *in vitro* biological activity (IC_50_ and pIC_50_ values) of the ligands compared with their respective docking scores from our calculations are listed in S2 Table. The first three compounds have an IC_50_ ranging from 50 to 250 μM, considered as the most potent compounds in this class. Compounds #4–7 with IC_50_ of 700 μM up to 1,100 μM represent blockers of average inhibition and finally the last two compounds (#8 and #9) are considered as weak blockers with 5,000 and 58,000 μM activity. Next, a Pearson correlation coefficient was computed to assess the relationship between the scores calculated from the docking poses of the ligands against the KCNQ1/KCNE1 protein complexes, and the pIC_50_ values of the compounds. There was a strong, positive correlation (r_pearson_ = 0.75) between the two variables, indicating that our model has been successful in discriminating blockers of variable activity. A scatter plot in Fig 8a summarizes the results.

To study the interactions of the ligands and their modes of binding within the pore of the channel, we chose representatives of ligands with different ranges of activity. Fig 8b shows the zoomed-in placement of these ligands within the binding site while interacting with the protein residues. Ligand #2 possesses a trifluoro-butoxy substitution at the 6-position on the aromatic ring. This bulky side chain interacts with the residues in the centre of the pore, underneath the selectivity filter. Substantial interactions of Ligand #2 with the binding site residues include Phe340, Ile337, and Thr312. The sulfonyl group makes contacts with Pro343, while the trifluoro group interacts with Phe340, Ala336 and Ile337. This mode of binding is consistent with the general mode of interaction of chromanols within the KCNQ1/KCNE1 ion channel [44]. Furthermore, the protrusion of the lengthy side chain of the molecule towards the centre of the pore can explain its high blocking activity. This extension is also responsible for the interaction of the ligand with potassium ions as will be discussed in the next section of ion permeation.

We also investigated the binding mode of Ligand #8 and #9 as representatives possessing weak blocking activity (See Fig 8b). This investigation helped us understand the main reason behind the large difference in their IC_50_ values compared to ligand #1–3. Both ligands can slightly fit within the binding site pocket, albeit with an inclination away from the pore and the selectivity filter. Ligand #8, for example, makes minimal contacts with the Thr312 residues of the different subunits, which are all located right below the selectivity filter. However, the ethyl group that is attached to the sulfonyl residue of the ligand has a tendency to interact with residues that are not central to the axis of the pore, e.g. Gly345. As seen in Fig 8b, these two ligands have cyanide substitutions at the 6-position of the aromatic ring, which is substantially shorter and less bulkier than those of the strong blockers (ligands #1, #2 and #3). Refer to Fig 6 for structures of the ligands.

Also, it is evident that increasing the size of the sulfonyl residue has a direct effect on the potency of the compounds, as is the case with #9, possessing a butyl substitution extending away from the sulfonyl group. The latter effect can be explained by the fact that the butyl entity interacts with residues on the periphery of the pore and thereby pulls the ligand away from its binding site. This hinders the physical presence of the drug molecule in the pore. Furthermore, contrary to ligand #2, the 6-position substitution in #8 and #9, i.e. the cyanide group is facing away from the pore and extends towards the opposite direction. This reduces the blockage of the ion passage and can also be a second reason behind their reduced potency.

In addition, the interaction of one ligand #4, which has a slightly lower potency compared to #1–3 (IC_50_ = 700 μM), was investigated (See Fig 8b). This compound also possesses a short substitution (a single fluorine group) at the 6-position of the benzene ring, similar to #8 and #9. However, compared to #8 and #9, it is more inclined towards the pore. This is clear from the contact it makes with Thr312, which is placed at the mouth of the selectivity filter. This interaction is completely absent in #8 and #9 as their cyanide group substitution is facing away from this residue. The ligand also makes contacts with Thr312, Ile337 and Phe340 which are all amongst the binding site residues indicating that it is central to the binding site cavity, right below the selectivity filter. However, the fluorine substitution does not occupy as much space as the side chain of Ligand #1 and #2 and therefore, its lower potency compared to the strong blockers may be justified in this way.

Overall, the results from the docking simulations enabled us to confirm the structure activity relationship of the Chromanol blockers and the reason behind their differential activity. The substitutions at the 6-position on the aromatic ring is the first determinant of potency. This substitution, depending on its size and direction of extension towards the central axis of the pore, can have differential effect on the conduction of the potassium ions. Secondly, the sulfonyl residue substitution also affects the potency of the compounds. This effect may be produced because the substitutions at this position can have an affinity to interact with the residues on the periphery of the pore. This interaction draws the drug molecule further away from the pore and thereby reducing their ability to produce physical blockage. This effect was clear in ligand #9, which has a butyl group extending from the sulfonyl entity. The encouraging success of the model in predicting the activity and blockage capacity indicated that the model is capable of predicting the correct binding mode and the interaction of the ligands with the channel. Thereby, this adds one more validation measure for our model with regards to its ability for predicting the off-target interactions of other drugs. Also, given the acceptable results we obtained from the docking studies, we decided to take our research question to the next stage, i.e. testing the effect of drugs on the potassium ion permeation (presented below).

### Effect of blockers on ion permeation: SMD simulations

One of the objectives in this work was to also understand how the binding of drugs onto the channel pore would affect the passage of ions through the KCNQ1/KCNE1 complex. To carry out this study, we decided to focus on 6 compounds, marked with asterisks in S2 Table. The compounds were selected such that they represent compounds with different ranges of activity. For instance, compounds #1, #2, #4 and #6 represented the group of strong-average inhibition. On the other hand, compounds #8 and #9 were possibly weak blockers of the KCNQ1 channel, as their IC_50_ values were 5,000 nM and 58,000 nM, respectively. The best ranking pose of the each of the ligand-channel complex were obtained from the docking calculations and optimized using short 4ns long MD simulations. For this purpose, the ligand-bound KCNQ1/KCNE1 complexes underwent a series of classical treatments as follows: Two-stage energy minimization → Heating → short NPT equilibration → ~4 ns long production MD simulation. Each of these steps were carried out using the parameters and the simulation setups as explained in the ‘Methods’ section.

**Fig 9:**
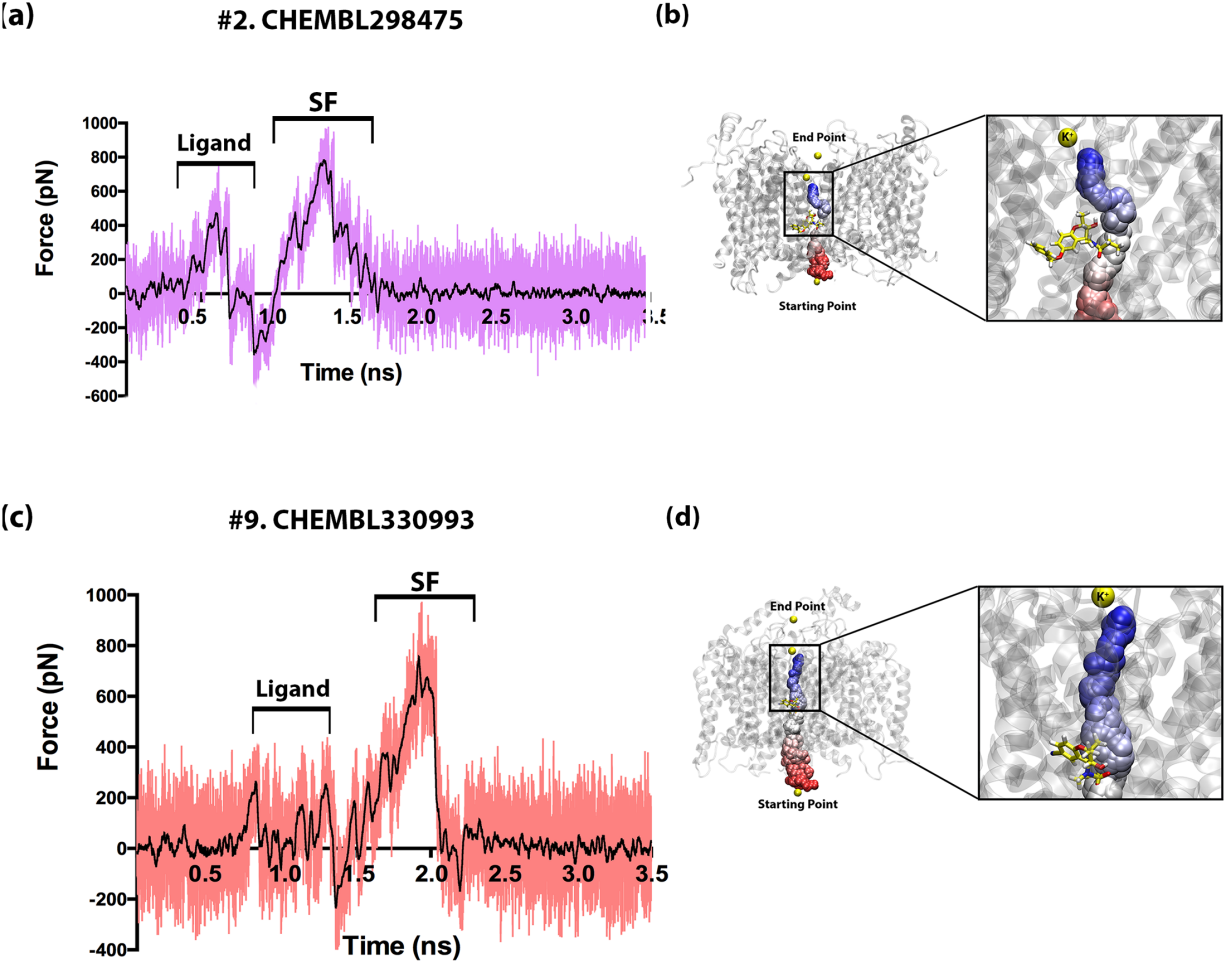
Force profiles for the pulling of a potassium ion through the ligand-bound KCNQ1/KCNE1 complex during the SMD simulations. The force profiles and the ion permeation processes of a strong channel blocker, or Ligand #2 (a-b), and a weak blocker, or Ligand #9 (c-d) are shown.

The equilibrated ligand-bound KCNQ1/KCNE1 complexes were then subjected to SMD simulations, as described earlier, wherein a single potassium ion was pulled from the intracellular region, through the pore, to the exterior of the channel. Comparisons of the resultant force profiles from the ligand-bound KCNQ1/KCNE1 complexes against those obtained for the ligand-free KCNQ1/KCNE1 complex and un-complexed KCNQ1 channel should, theoretically, be useful to reveal the effects of ligand-binding on the potassium ion permeation through the channel complex. Fig 9 compares the force profiles of the SMD simulations performed on a strong blocker (ligand #2)-channel complex (a) and a weak blocker (ligand #9)-channel complex (b). The force profiles for the other ligand-channel complexes are provided in the supplementary information, S3 Fig. In an overall, it is interesting to note that the peaks corresponding to the release of ions from the selectivity filter residues (as marked in Fig 9) are almost similar to those seen in the ligand-free systems (see in Figs 2 and 3). The number of peaks related to these regions and their corresponding intensities are all almost similar in all the three types of systems studied in this work, i.e. uncomplexed KCNQ1, KCNQ1/KCNE1 complex, ligand-bound KCNQ1/KCNE1 complexes. This describes that neither protein-protein interactions nor the ligand-binding affects the selectivity filter in the KCNQ1 ion channel significantly. And in all cases, the release of the potassium ion from the selectivity filter residues remains a significant rate-limiting stage in ion permeation and this process costs ~600 pN force in all our SMD simulations. To explain the SMD results in more details, the force profiles for the systems encompassing ligand #2 (strong-blocker) and ligand #9 (weak-blocker) are shown in Fig 9a and 9b. In case of #2, the specific effects of ligand binding on the force profile from the SMD simulations are captured during the initial stage of simulation. This peak mostly overlaps with the peaks for BA1-BA2 sites seen for KCNQ1/KCNE1 complex. This is because, the sites BA1 and BA2 form a part of the ligand binding site in the KCNQ1 channel, which is populated with Phe340, Thr312 and Ile337. Hence, the ligands are bound near these sites in the channel complex. As a consequence, the peaks in the SMD force profile corresponding to pulling the ion from the ligand-barrier (in Fig 9) are concentrated at the initial stage of time-scale, which mostly coincides to the peak seen in KCNQ1/KCNE1 complex (Fig 3). However, the intensity of this peak in the case of ligand-bound systems increases as much as 200 pN. This indicates that the binding of a ligand at this site enhances the blockade on the ion permeation pathway, thereby, necessitating much higher force (> 500 pN, as shown in Fig 9a) for ion release during the SMD simulations. The same trend is observed for all other strong blockers in this study (S3 Fig).

Furthermore, our SMD simulations were able to discern the high-affinity blockers from those of low-affinity ones. The force profile for Ligand #9, representing one of the weak ligands is shown in Fig 9b. The major difference in the force profiles of the high-affinity and weak-affinity blockers are observed in the first peak (marked as Ligand on the force profile plots) again. The intensity of the first peak is as high as ~500–600 pN for the high-affinity blockers (#1, #2, #4, #6 in S3 Fig). While this peak corresponds to the ligand-binding region, the constriction is mainly caused by the respective side chain groups of drugs, which interact strongly with the surrounding residues and restrict the smooth ion movement (Fig 9b and 9c). For example, in the ligand #2-bound KCNQ1/KCNE1 complex, the ligand has much longer functional group which obstructs the ion passage. Particularly, the sulfonyl group present in this ligand renders a strong electrostatic environment for the passing ion, thus requiring much higher external force to release the ion (Fig 9b). On the other hand, the weak-affinity ligands in this study bind in an orientation that does not cause major hindrance for the ion permeation. For instance, when bound to the KCNQ1/KCNE1 complex, the cyano group substitution of the aromatic ring in ligand #9 is drifted away from the ion passage (refer to Fig 9d) and, as a result, pulling the ion across this ligand did not require much force. Hence, the force profile for this complex with ligand #9 does not show any high-intensity peak < 1.5 ns. Pore dimension analyses (S7 Fig) on the drug-bound systems also supported the findings from SMD simulations. It was found that the pore dimensions in the high-affinity binders are much wider; whereas, the pore radius of the weak-affinity ligand systems are less constricted allowing much easier ion permeation. Hence, our study has not only been able to provide mechanistic insights into ion permeation in KCNQ1, in the presence and absence of protein-protein interactions and ligand binding, but also discriminate the strong blockers from the weak ones. Further experimental testing on the findings from this work are warranted.

## Conclusions

In this study, we described the effects of KNCQ1-KCNE1 interactions and the small-molecule binding on the ion permeation mechanisms through the KCNQ1 channel, using atomistic and steered MD simulations. The 3D structures of the open-state of the unbound-KCNQ1 and the KCNQ1/KCNE1 complex were initially equilibrated using long-scale MD simulations, which revealed that the complex form was more stable than that of the unbound-KCNQ1 channel. SMD simulations were performed on the structures of KCNQ1 and KCNQ1/KCNE1 complex (collected from the MD trajectories), during which a potassium ion was pulled from the intracellular region to the extracellular bulk water through the KCNQ1 channel. The SMD simulations revealed that the selectivity filter residues formed the only high-energy barrier in the unbound KCNQ1 structure. While this high-energy barrier still existed in the KCNQ1/KCNE1 complex, there were a couple of additional energetic barriers found during the early stages of SMD simulations. Analyses of the SMD trajectories of the KCNQ1/KCNE1 complex revealed that the inter-protein interactions from KCNE1 had constricted the pore in KCNQ1, which resulted in two small energetic barriers caused by residues, (1) Ser349, Ala344, Gly345; and (2) Thr312, Ile337 and Phe340, along the ion permeation pathway. This explains the possible molecular mechanisms underpinning the slow channel activation in the KCNQ1/KCNE1 complex observed in previous experiments. Binding of small-molecule blockers of Chromanol 293B derivatives onto the KCNQ1/KCNE1 complex only enhanced these additional peaks seen in the ligand-free KCNQ1/KCNE1 complex. Nevertheless, the effects were less pronounced (in terms of the required force for ion pulling), when weak-blockers were bound in the complex. While KCNE1 interactions and small-molecule binding affected the ion permeation into the pore of the channel, they did not impact the selectivity filter residues in KCNQ1. In fact, the release of the ion from the selectivity filter barrier always remained the most significant rate-limiting step (requiring the largest amount of external force) in all the complexes in this study. Thus, our study provides some qualitative and quantitative insights into the effects of protein-protein interactions and small-molecule binding on the ion permeation processes in KCNQ1, an important voltage-gated ion channel in the heart. The findings presented here will have some implications in understanding the potential off-target interactions of the drugs with the KCNQ1/KCNE1 channel that lead to cardiotoxic effects.

## Supporting information

S1 Table List of known LQTS1-associated single-point mutations in human KCNQ1 channel. (PDF)

S2 Table Ranking of the ligands by their pIC_50_s compared with their IC_50_ values and docking scores. The docking score in the table is the average from 3 scoring functions: AutoDock Vina, NNScore 2.0 and RF-Score-VS. * Ligands used for ion permeation studies.
(PDF)

S1 Fig Lone KCNQ1 SMD repeats. (TIF)

S2 Fig KCNQ1/KCNE1 SMD repeats. (TIF)

S3 Fig The force profiles of the SMD repeats for the selected ligand-bound KCNQ1/KCNE1 complexes (#1, #2, #4, #6, #8 and #9). (TIF)

S4 Fig The backbone RMSD graphs of the proteins in the four systems during SMD simulations. For KCNQ1, KCNQ1/KCNE1, and KCNQ1/KCNE1 system bound to Strong blocker (#1) and Weak blocker (#9).
(TIF)

S5 Fig Movement of water through the pore of the KCNQ1 channel. The water molecules are colored from red to blue based on the simulation timestep. The protein is shown in cartoon presentation.
(TIF)

S6 Fig The dimensions of the pore shown in surface representation. (a) KCNQ1 without KCNE1 before and after MD, (b) KCNQ1 in complex with KCNE1 before and after MD. Color code: Red is where the pore radius is too tight for a water molecule. Green where there is room for a single water molecule. Blue is where the radius is double the minimum for a single water molecule.
(TIF)

S7 Fig The dimensions of the pore (shown in surface representation) in KCNQ1/KCNE1 systems with the 6 docked ligands (shown in purple color). *Pore colour code: Red is where the pore radius is too tight for a water molecule. Green where there is room for a single water molecule. Blue is where the radius is double the minimum for a single water molecule.
(TIF)
