# Supplementary material for: Effects of protein-protein interactions and ligand binding on the ion permeation in KCNQ1 potassium channel
Source: PLoS One. 2018 Feb 14;13(2):e0191905. doi: 10.1371/journal.pone.0191905 (PMC5812580; doi:10.1371/journal.pone.0191905)
Supplement: S1 Table — (PDF) [file pone.0191905.s001.pdf]

S1 Table. List of known LQTS1-associated single-point mutations in human KCNQ1 channel:

(1) List of single-point mutations in KCNQ1 channel with unknown pathological significance

A2V[1]; P7S[1]; A46V[1]; S66F[1]; P73T[1-2]; Y111C[1]; E115G[2]; P117L[1]; C122Y[2]; F127L[1]; V133I[2]; L134P[1]; C136F[2]; L137F[3]; T144A[1]; E146K[3]; T153M[1]; F157C[4]; E160K[2]; V162M[1]; G168R[1-2]; V172M[1]; V173D[3]; R174H[1-2]; R174P[1]; A178P; A178T;[1, 5] G179S[1]; Y184H[1] Y184S[6]; G186R[1]; G189R[6]; R190L[1] R190Q[1-2] R190W[3]; L191P[7]; R192P[3]; A194P; R195W[1]; I198V[1]; S199A[1]; D202H[3]; I204F[2] I204M[1, 3]; S209F[3]; V215M[1, 3]; G216R[8]; T224M[1]; S225L[1-2]; R231C R231H[1, 3]; I235N[1-2]; L239P[3]; V241G[1]; V254L[3]; H258N[3] H258R[3]; R259C[9] R259H[1-2]; L262V[1, 3]; L266P[1-2]; I268S[1]; G269D[1-2] G269S[1-2]; G272S[3]; L273R[2]; I274V[1]; F275S[7]; S277P[1]; Y278H[2]; V280E[1, 3]; Y281C[1]; L282P[1]; A283G[1]; A287E[3]; E290K[2]; G292D[1-2]; R293C[1-2]; A300T; A302E[1]; L303P[3]; W304R[2]; W305R[1]; G306R[1]; V308D[3]; T309R; V310I; I313M[10]; G314C[1]; Y315C[2]; Y315S[2]; G316E[2]; G316R[2]; G316V[1]; G325R[1, 10]; F339Y[1]; A341E[1]; A341G[1]; A341V[1-2]; L342F[1]; P343L[1, 3] P343R[3]; P343S[2]; A344E[2]; A344V[2]; S349W[2]; G350R[3]; F351S[1, 3]; L353P[2]; K354R[1]; R360M[1]; R360T[3]; K362R[1-2]; N365H[1]; R366P[10]; R366Q[1]; R366W[1-2]; A371T; A372D[3]; S373P; L374H[1]; W379G[1]; R380S[2]; E385K[1]; S389P[1]; S389Y[2]; T391I; T391T[1]; W392R; K393M[3]; R397W[1]; K398R[1]; V417M; D446E[1]; P448L[1]; P448R; R451W[1]; R452W; G460S[1]; P477L[1]; R511W[1]; R518G[3]; R518P[3]; R518Q[1]; M520R[1]; Y522S[1]; V524G[1-2]; A525T[1]; A525V[1]; K526E[2]; V541I[1]; E543K[1]; Q547R[1]; G548D[3]; V554A[3]; S566Y[1-2]; G568R[1-2]; K569E[1]; S571L[1]; F573L[3]; R583H[3]; T587M r[1-2]; R591C[1]; R591H[1-2]; R594P[1-2]; R594Q[1-2]; E596K[1]; T600M[1]; D611N[1]; G626S[1-2]; G635R[1];

(2) List of single-point mutations in KCNQ1 channel with known properties

Site	Mutation	Known property
242	D242N[1, 10-11]	Reduction in outward K+ current and in plasma membrane localization
243	R243C[1-2]	Slower activation rate and voltage-dependent activation/inactivation shifted to positive potentials. Mutated channels are non-fuctional.
243	R243P[9, 12]	Complete loss of outward K+ current.
248	W248R[13]	Slower activation rate and voltage-dependent activation/inactivation shifted to positive potentials. Mutated channels are non-fuctional.
250	L250H[11-12]	Complete loss of outward K+ current.
261	E261K[13]	Loss of channel activity
277	S277L[1-2, 7]	Loss of function and mutation acts as a dominant-negative manner.
306	G306V[7, 12]	Complete loss of outward K+ current.
311	T311I[12, 14]	Impaired outward K+ current.
312	T312I[1-2, 5]	Loss of channel activity
317	D317N[12, 14]	Complete loss of outward K+ current when expressed alone.

320	P320A[1]	Loss of function and mutation acts as a dominant-negative manner.
322	T322M[1, 3, 12]	Impaired outward K+ current.
345	G345R[6]	Familial sudden death
533	R533W[1]	Positive voltage shift of the channel activation;
539	R539W[1-2]	Positive voltage shift of the channel activation;
546	S546W[1-2]	Decreases the interaction with KCNE1; reduces IKS current density.
555	R555C[1-2]; R555H[1]	Decreases the interaction with KCNE1; reduces IKS current density.
557	K557E[1]	slows activation kinetics; accelerates deactivation kinetics; affects interaction with KCNE1
586	N586D[12]	Reduces outward K+ current.
589	G589D[12]	Strongly affects the K+ current.
590	A590T[2]	Reduces Iks density and causes a right-shift of the current voltage.
619	L619M[2, 12]	Reduces outward K+ current.

References:

1. Kapplinger, J. D.; Tester, D. J.; Salisbury, B. A.; Carr, J. L.; Harris-Kerr, C.; Pollevick, G. D.; Wilde, A. A.; Ackerman, M. J., Spectrum and prevalence of mutations from the first 2,500 consecutive unrelated patients referred for the FAMILION long QT syndrome genetic test. *Heart rhythm* **2009,** *6* (9), 1297-303.
2. Tester, D. J.; Will, M. L.; Haglund, C. M.; Ackerman, M. J., Compendium of cardiac channel mutations in 541 consecutive unrelated patients referred for long QT syndrome genetic testing. *Heart rhythm* **2005,** *2* (5), 507-17.
3. Napolitano, C.; Priori, S. G.; Schwartz, P. J.; Bloise, R.; Ronchetti, E.; Nastoli, J.; Bottelli, G.; Cerrone, M.; Leonardi, S., Genetic testing in the long QT syndrome: development and validation of an efficient approach to genotyping in clinical practice. *Jama* **2005,** *294* (23), 2975-80.
4. Larsen, L. A.; Christiansen, M.; Vuust, J.; Andersen, P. S., High-throughput single-strand conformation polymorphism analysis by automated capillary electrophoresis: robust multiplex analysis and pattern-based identification of allelic variants. *Human mutation* **1999,** *13* (4), 318-27.
5. Shalaby, F. Y.; Levesque, P. C.; Yang, W. P.; Little, W. A.; Conder, M. L.; Jenkins-West, T.; Blanar, M. A., Dominant-negative KvLQT1 mutations underlie the LQT1 form of long QT syndrome. *Circulation* **1997,** *96* (6), 1733-6.
6. Jongbloed, R. J.; Wilde, A. A.; Geelen, J. L.; Doevendans, P.; Schaap, C.; Van Langen, I.; van Tintelen, J. P.; Cobben, J. M.; Beaufort-Krol, G. C.; Geraedts, J. P.; Smeets, H. J., Novel KCNQ1 and HERG missense mutations in Dutch long-QT families. *Human mutation* **1999,** *13* (4), 301-10.
7. Liu, W.; Yang, J.; Hu, D.; Kang, C.; Li, C.; Zhang, S.; Li, P.; Chen, Z.; Qin, X.; Ying, K.; Li, Y.; Li, Y.; Li, Z.; Cheng, X.; Li, L.; Qi, Y.; Chen, S.; Wang, Q., KCNQ1 and KCNH2 mutations associated with long QT syndrome in a Chinese population. *Human mutation* **2002,** *20* (6), 475-6.
8. van den Berg, M. H.; Wilde, A. A.; Robles de Medina, E. O.; Meyer, H.; Geelen, J. L.; Jongbloed, R. J.; Wellens, H. J.; Geraedts, J. P., The long QT syndrome: a novel missense mutation in the S6 region of the KVLQT1 gene. *Human genetics* **1997,** *100* (3-4), 356-61.
9. Millat, G.; Chevalier, P.; Restier-Miron, L.; Da Costa, A.; Bouvagnet, P.; Kugener, B.; Fayol, L.; Gonzalez Armengod, C.; Oddou, B.; Chanavat, V.; Froidefond, E.; Perraudin, R.; Rousson, R.; Rodriguez-Lafrasse, C., Spectrum of pathogenic mutations and associated polymorphisms in a cohort of 44 unrelated patients with long QT syndrome. *Clinical genetics* **2006,** *70* (3), 214-27.
10. Tanaka, T.; Nagai, R.; Tomoike, H.; Takata, S.; Yano, K.; Yabuta, K.; Haneda, N.; Nakano, O.; Shibata, A.; Sawayama, T.; Kasai, H.; Yazaki, Y.; Nakamura, Y., Four novel KVLQT1 and four novel HERG mutations in familial long-QT syndrome. *Circulation* **1997,** *95* (3), 565-7.
11. Itoh, T.; Tanaka, T.; Nagai, R.; Kikuchi, K.; Ogawa, S.; Okada, S.; Yamagata, S.; Yano, K.; Yazaki, Y.; Nakamura, Y., Genomic organization and mutational analysis of KVLQT1, a gene responsible for familial long QT syndrome. *Human genetics* **1998,** *103* (3), 290-4.
12. Mousavi Nik, A.; Gharaie, S.; Jeong Kim, H., Cellular mechanisms of mutations in Kv7.1: auditory functions in Jervell and Lange-Nielsen syndrome vs. Romano-Ward syndrome. *Frontiers in cellular neuroscience* **2015,** *9*, 32.
13. Franqueza, L.; Lin, M.; Shen, J.; Splawski, I.; Keating, M. T.; Sanguinetti, M. C., Long QT syndrome-associated mutations in the S4-S5 linker of KvLQT1 potassium channels modify gating and interaction with minK subunits. *The Journal of biological chemistry* **1999,** *274* (30), 21063-70.
14. Saarinen, K.; Swan, H.; Kainulainen, K.; Toivonen, L.; Viitasalo, M.; Kontula, K., Molecular genetics of the long QT syndrome: two novel mutations of the KVLQT1 gene and phenotypic expression of the mutant gene in a large kindred. *Human mutation* **1998,** *11* (2), 158-65.
