# Supplementary material for: Effects of protein-protein interactions and ligand binding on the ion permeation in KCNQ1 potassium channel
Source: PLoS One. 2018 Feb 14;13(2):e0191905. doi: 10.1371/journal.pone.0191905 (PMC5812580; doi:10.1371/journal.pone.0191905)
Supplement: S2 Table — The docking score in the table is the average from 3 scoring functions: AutoDock Vina, NNScore 2.0 and RF-Score-VS. * Ligands used for ion permeation studies. (PDF) [file pone.0191905.s002.pdf]

S2 Table. Ranking of the ligands by their pIC_{50}s compared with their IC_{50} values and docking scores. The docking score in the table is the average from 3 scoring functions: AutoDock Vina, NNScore 2.0 and RF-Score-VS. * Ligands used for ion permeation studies.

**Compound #**	**CHEMBL _ID**	**Docking Score**	**IC_{50} (nM)**	**pIC_{50}**
1 *	CHEMBL124454	7.216	50	7.3
2 *	CHEMBL298475	7.144	120	6.9
3	CHEMBL125307	7.083	250	6.6
4 *	CHEMBL340025	6.654	700	6.2
5	CHEMBL124810	7.003	900	6.1
6 *	CHEMBL338171	6.268	1100	6.0
7	CHEMBL125259	6.404	3100	5
8 *	CHEMBL434045	6.763	5000	5.3
9 *	CHEMBL330993	6.362	58000	4.2
