# Supplementary figures and images for: Effects of protein-protein interactions and ligand binding on the ion permeation in KCNQ1 potassium channel
Source: PLoS One. 2018 Feb 14;13(2):e0191905. doi: 10.1371/journal.pone.0191905 (PMC5812580; doi:10.1371/journal.pone.0191905)

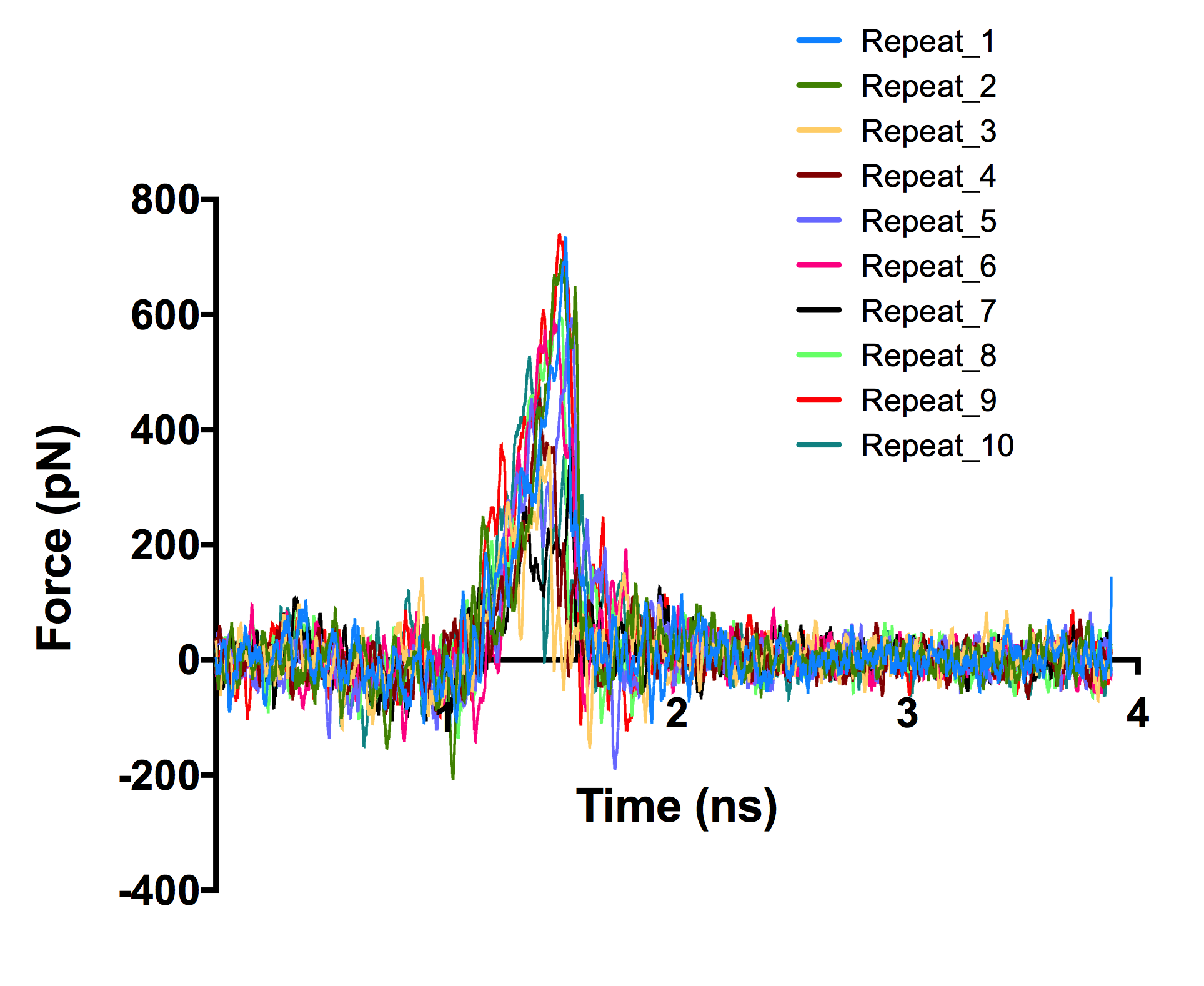

Supplement: S1 Fig — (TIF) [file pone.0191905.s003.tif]

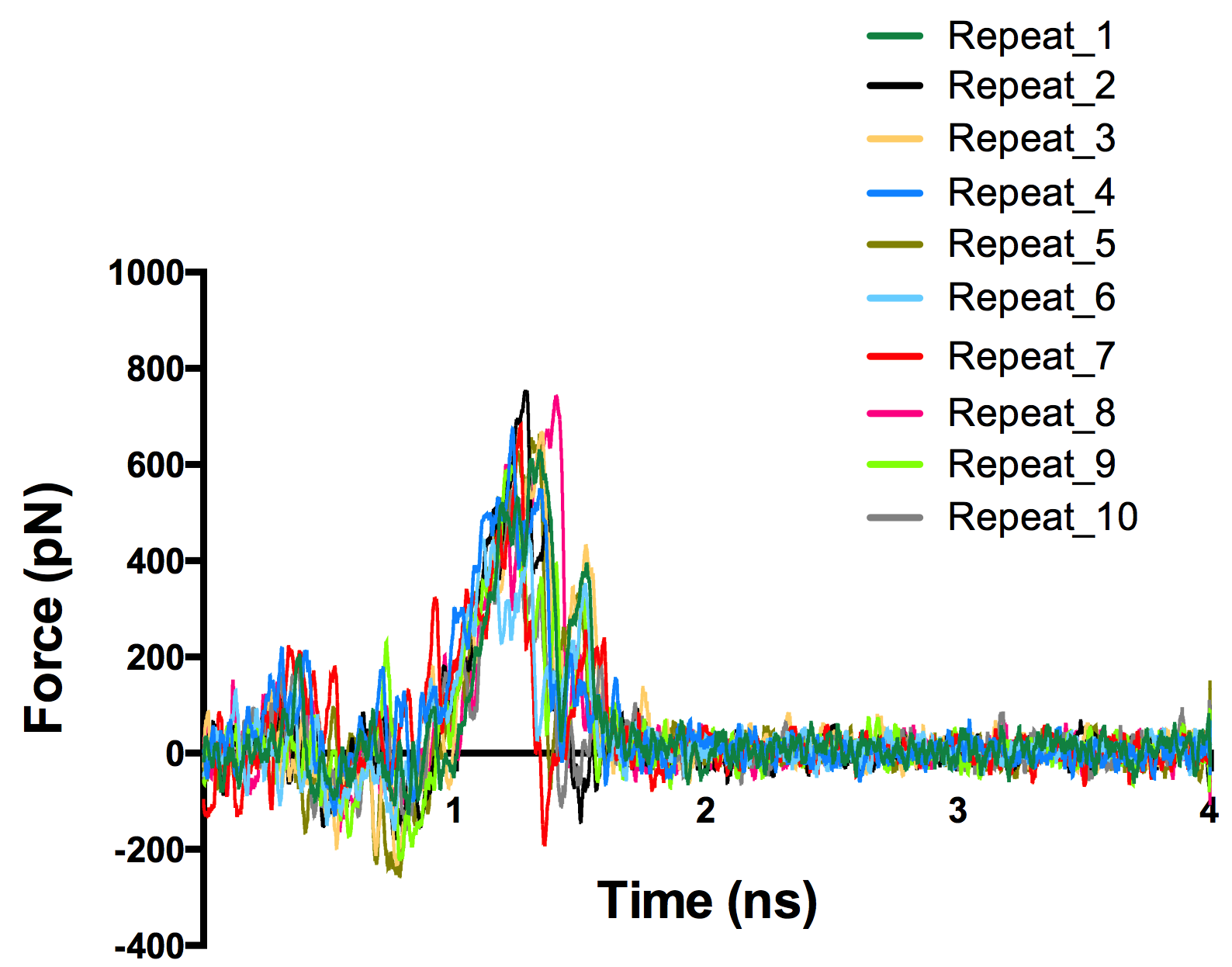

Supplement: S2 Fig — (TIF) [file pone.0191905.s004.tif]

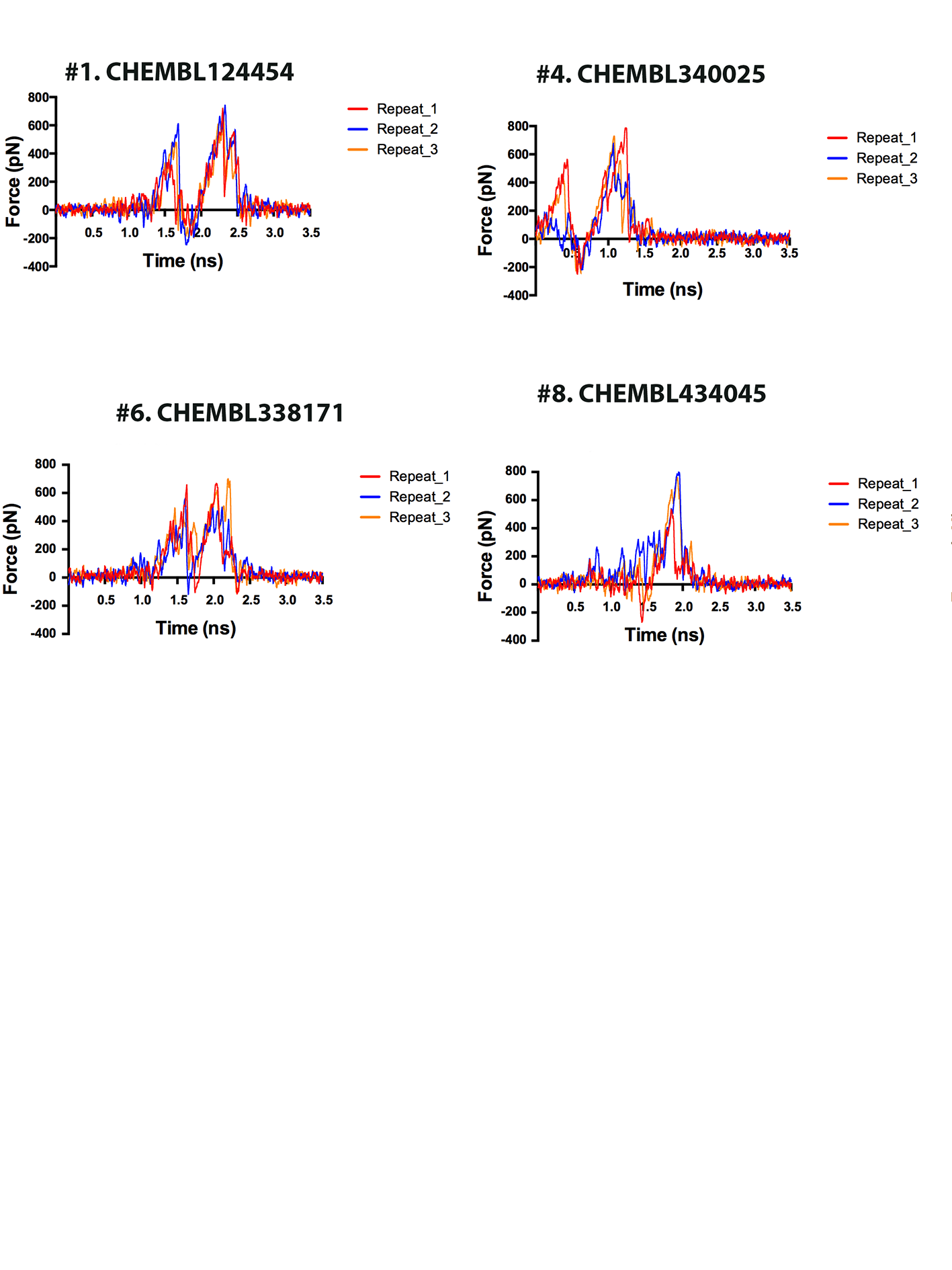

Supplement: S3 Fig — (TIF) [file pone.0191905.s005.tif]

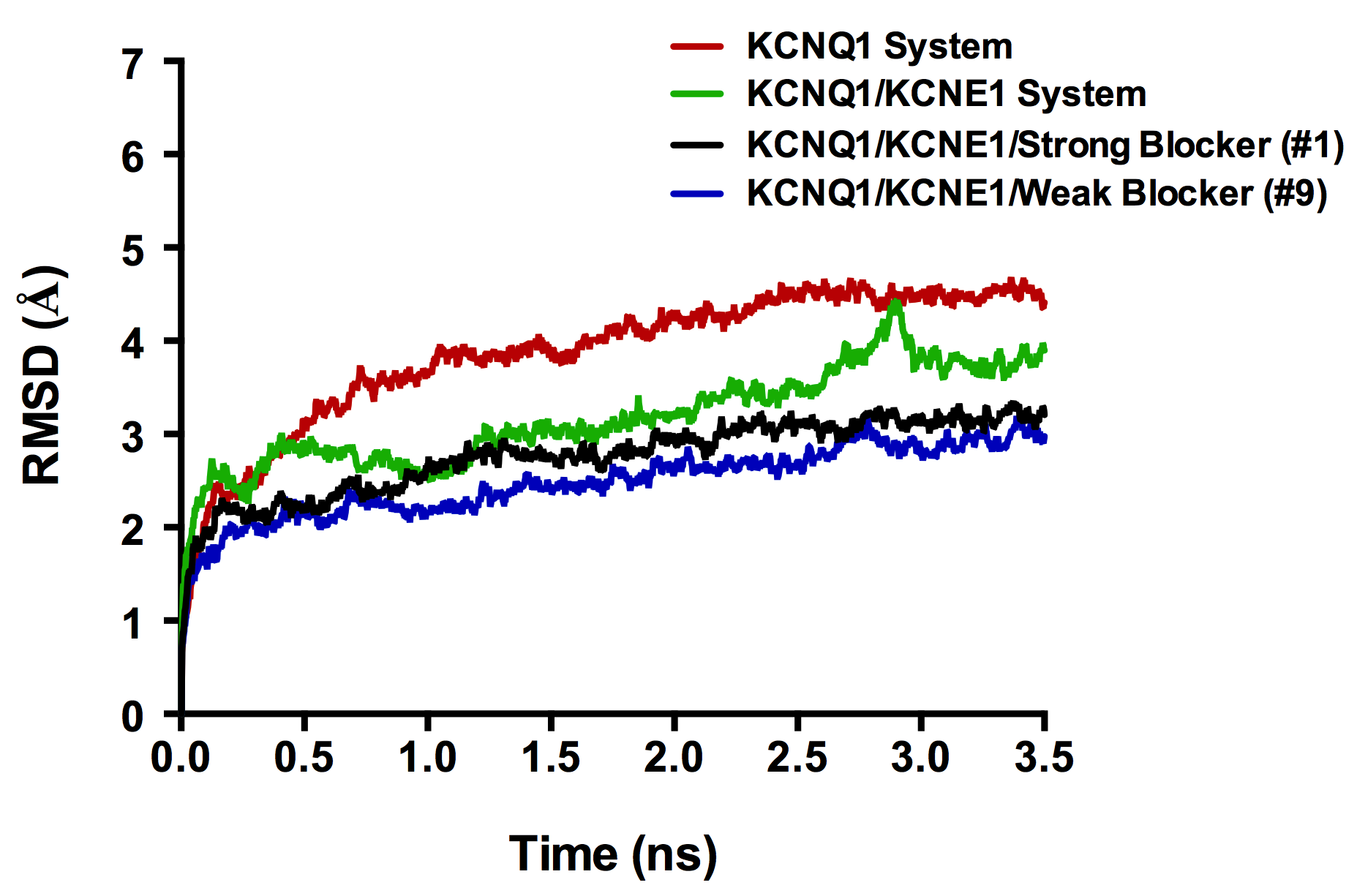

Supplement: S4 Fig — For KCNQ1, KCNQ1/KCNE1, and KCNQ1/KCNE1 system bound to Strong blocker (#1) and Weak blocker (#9). (TIF) [file pone.0191905.s006.tif]

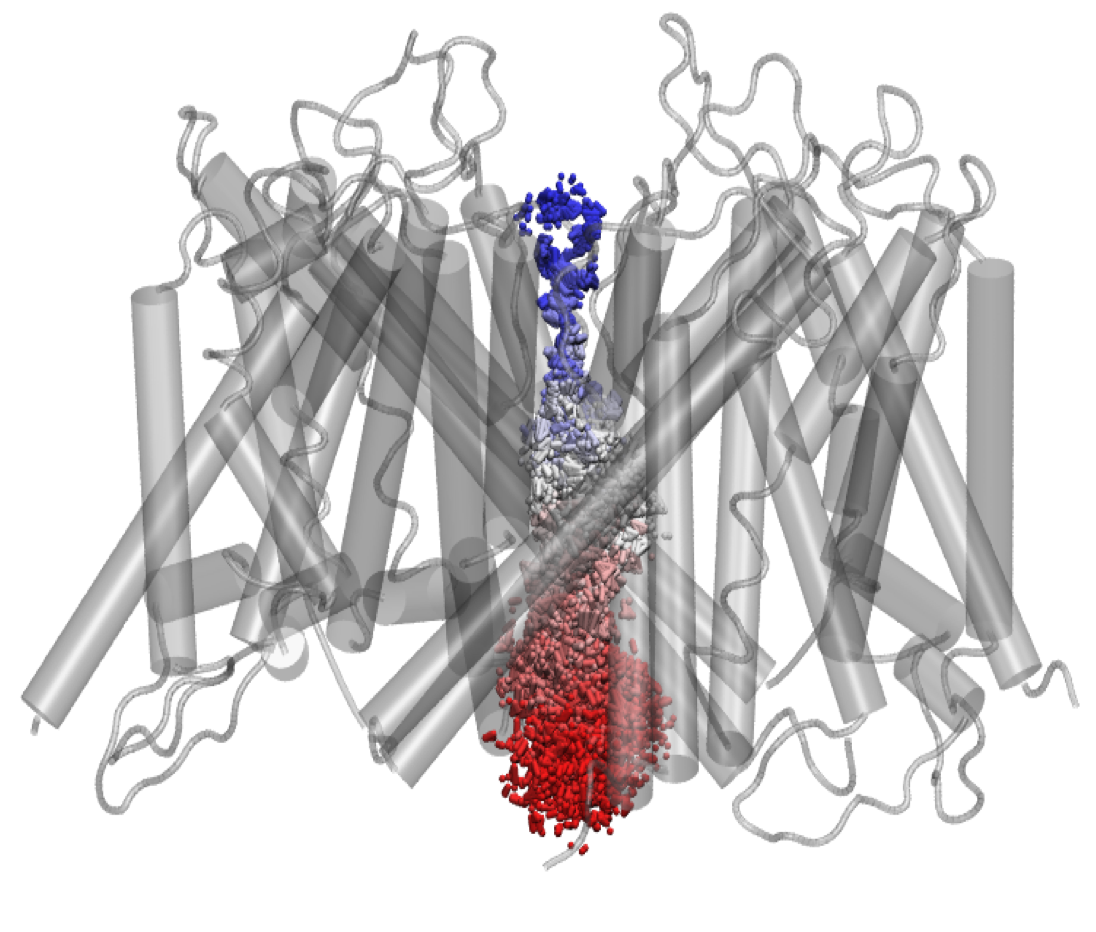

Supplement: S5 Fig — The water molecules are colored from red to blue based on the simulation timestep. The protein is shown in cartoon presentation. (TIF) [file pone.0191905.s007.tif]

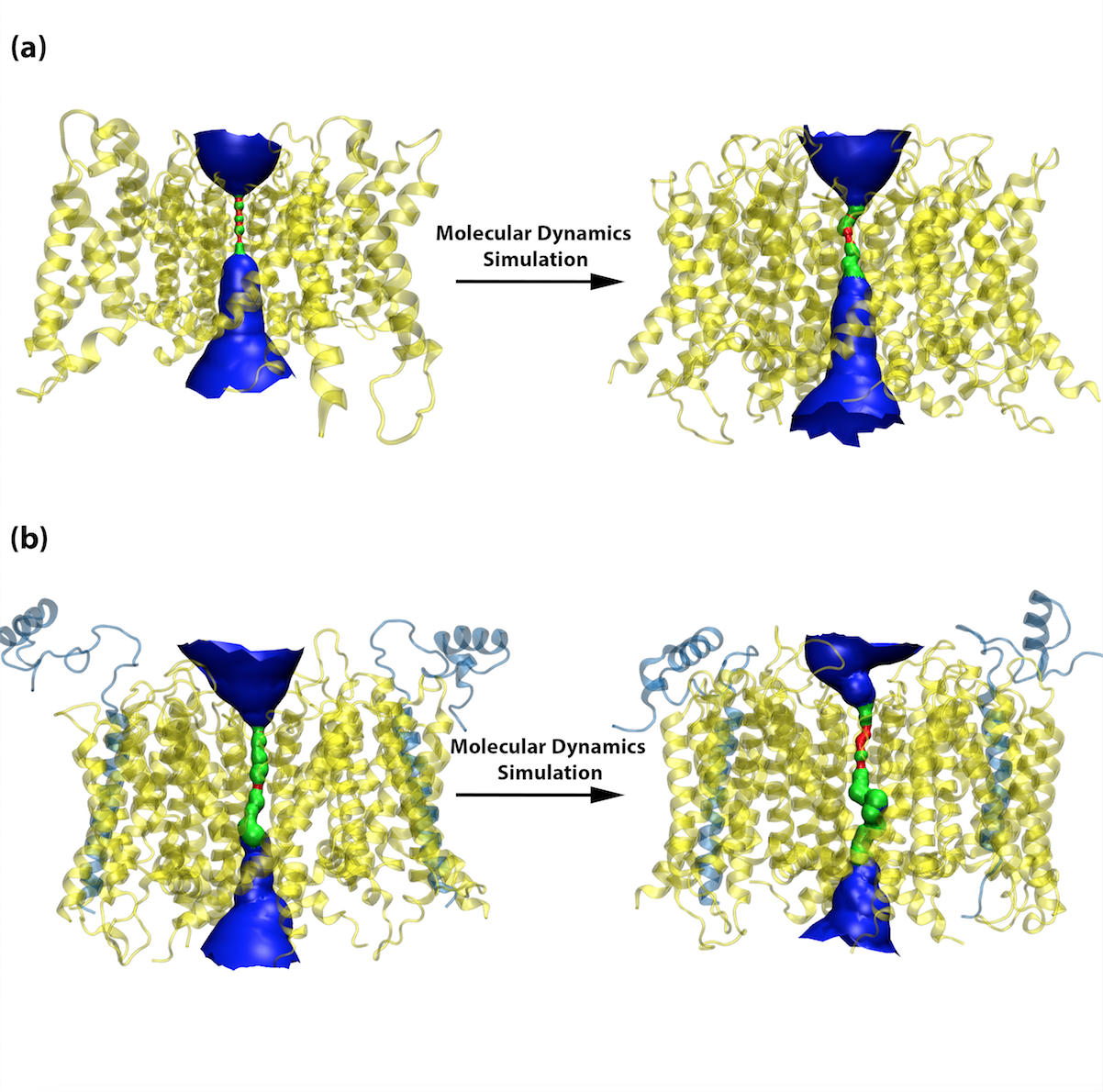

Supplement: S6 Fig — (a) KCNQ1 without KCNE1 before and after MD, (b) KCNQ1 in complex with KCNE1 before and after MD. Color code: Red is where the pore radius is too tight for a water molecule. Green where there is room for a single water molecule. Blue is where the radius is double the minimum for a single water molecule. (TIF) [file pone.0191905.s008.tif]

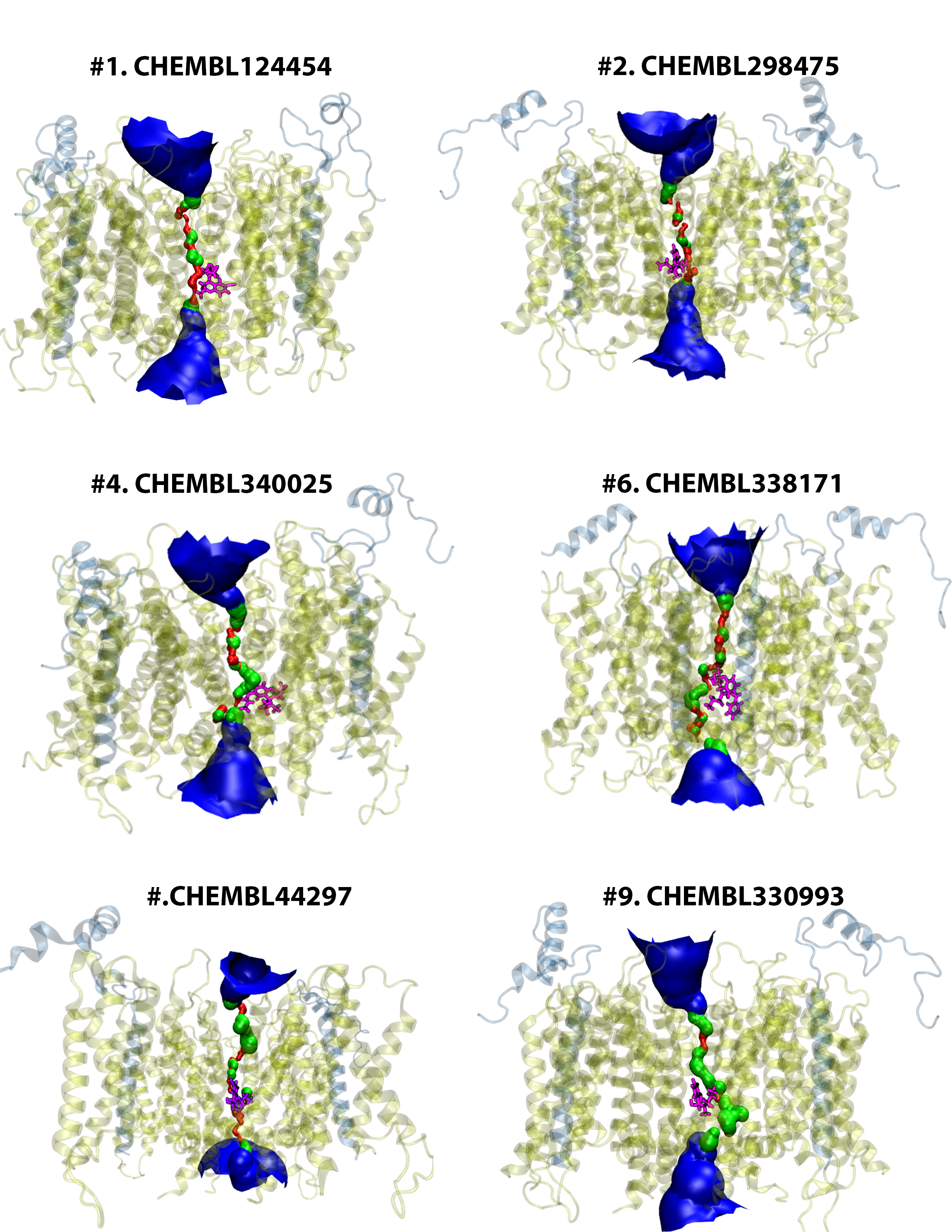

Supplement: S7 Fig — *Pore colour code: Red is where the pore radius is too tight for a water molecule. Green where there is room for a single water molecule. Blue is where the radius is double the minimum for a single water molecule. (TIF) [file pone.0191905.s009.tif]
